# Functional Characterization of FLT3 Receptor Signaling Deregulation in Acute Myeloid Leukemia by Single Cell Network Profiling (SCNP)

**DOI:** 10.1371/journal.pone.0013543

**Published:** 2010-10-27

**Authors:** David B. Rosen, Mark D. Minden, Steven M. Kornblau, Aileen Cohen, Urte Gayko, Santosh Putta, John Woronicz, Erik Evensen, Wendy J. Fantl, Alessandra Cesano

**Affiliations:** 1 Nodality, Inc., South San Francisco, California, United States of America; 2 Department of Medical Oncology/Hematology, The University of Toronto, Princess Margaret Hospital, Toronto, Ontario, Canada; 3 Department of Stem Cell Transplantation and Cellular Therapy, The University of Texas M. D. Anderson Cancer Center, Houston, Texas, United States of America; University of Hong Kong, China

## Abstract

**Background:**

Molecular characterization of the FMS-like tyrosine kinase 3 receptor (FLT3) in cytogenetically normal acute myeloid leukemia (AML) has recently been incorporated into clinical guidelines based on correlations between FLT3 internal tandem duplications (FLT3-ITD) and decreased disease-free and overall survival. These mutations result in constitutive activation of FLT3, and FLT3 inhibitors are currently undergoing trials in AML patients selected on FLT3 molecular status. However, the transient and partial responses observed suggest that FLT3 mutational status alone does not provide complete information on FLT3 biological activity at the individual patient level. Examination of variation in cellular responsiveness to signaling modulation may be more informative.

**Methodology/Principal Findings:**

Using single cell network profiling (SCNP), cells were treated with extracellular modulators and their functional responses were quantified by multiparametric flow cytometry. Intracellular signaling responses were compared between healthy bone marrow myeloblasts (BMMb) and AML leukemic blasts characterized as FLT3 wild type (FLT3-WT) or FLT3-ITD. Compared to healthy BMMb, FLT3-WT leukemic blasts demonstrated a wide range of signaling responses to FLT3 ligand (FLT3L), including elevated and sustained PI3K and Ras/Raf/Erk signaling. Distinct signaling and apoptosis profiles were observed in FLT3-WT and FLT3-ITD AML samples, with more uniform signaling observed in FLT3-ITD AML samples. Specifically, increased basal p-Stat5 levels, decreased FLT3L induced activation of the PI3K and Ras/Raf/Erk pathways, decreased IL-27 induced activation of the Jak/Stat pathway, and heightened apoptotic responses to agents inducing DNA damage were observed in FLT3-ITD AML samples. Preliminary analysis correlating these findings with clinical outcomes suggests that classification of patient samples based on signaling profiles may more accurately reflect FLT3 signaling deregulation and provide additional information for disease characterization and management.

**Conclusions/Significance:**

These studies show the feasibility of SCNP to assess modulated intracellular signaling pathways and characterize the biology of individual AML samples in the context of genetic alterations.

## Introduction

AML is associated with a wide range of genetic alterations, including mutations in receptor tyrosine kinases (RTKs) that perturb intracellular signaling networks which play a role in leukemia pathogenesis and are manifested in the clinical heterogeneity of the disease. For example, internal tandem duplications in the juxtamembrane region or tyrosine kinase 1 domains of the FLT3 RTK are reported to result in autonomous, ligand independent signaling with consequent increases in survival and proliferation [1]–[3]. FLT3-ITD mutations are among the most common somatic mutations in AML occurring in 20–35% of adult [4]–[10] and ∼5–15% of pediatric [11]–[13] AML. While the presence of FLT3-ITD is not predictive of response to conventional induction chemotherapy, the presence of FLT3-ITD has consistently been shown to confer a poor prognosis with significantly shorter disease-free and relapse-free survival. Thus, both the NCCN and European LeukemiaNet guidelines now recommend testing for FLT3-ITD at diagnosis to guide post-remission therapeutic selection after induction chemotherapy in patients with cytogenetically normal (CN) AML [5]–[8], [10]–[18].

The length of the DNA insertion that constitutes the ITD and the mutational load or allelic ratio of mutated to wild type (WT) FLT3 receptor vary among patient leukemia samples. Although length of ITD has not been consistently reported to be associated with clinical outcomes, higher levels of ITD mutational load have been associated with worse outcomes in multiple studies [5], [7], [19]–[22]. In this regard, analysis of FLT3-ITD mutational load could provide more useful information compared to FLT3 receptor mutational status alone, but it has yet to be included in the current treatment guidelines. Overall current FLT3 receptor molecular tests provide no information about the functional consequences of these mutations on intracellular signaling pathways and do not detect the presence of other functionally related mutations or alterations that may cause deregulation of FLT3 receptor pathway activity. Furthermore the presence of other molecular events such as nucleophosmin (NPM1), CCAAT/enhancer-binding protein alpha (CEBP/α), or RUNX1 modifies the prognostic impact of FLT3 receptor mutational status [23]–[29].

Many studies have provided insight into intracellular signaling pathways regulated by the FLT3 receptor system [30]–[37]. Upon binding FLT3L, the WT FLT3 receptor undergoes dimerization followed by tyrosine kinase activation, transphosphorylation of the receptor cytoplasmic domain and recruitment of SH2-domain containing adaptor proteins which activate downstream signal transduction pathways such as Ras/Raf/Map Kinase and PI3K. In contrast, in cell lines expressing FLT3-ITD mutation, these proteins [in addition to signal transducer and activator of transcription (Stat) 5], are constitutively phosphorylated [34]–[36], [38]–[40]. Of relevance, increased basal levels of p-Stat5 AML blasts were shown to be predictive of the presence of FLT3-ITD mutations in patient samples [41].

Single cell network profiling (SCNP) using multiparameter flow cytometry is a distinct proteomic platform for analyzing and interpreting protein expression and pathway activity under baseline and modulated conditions. Using viable cells, measurements of endogenous proteins in relevant signaling pathways are made before and after *in vitro* exposure to modulators such as growth factors, cytokines or therapeutic agents known to be important for myeloid biology and clinical application (Table 1, Table S1). SCNP interrogates the physiology of signaling pathways in single cells by measuring network properties not apparent in resting cells (e.g. failure of a specific pathway to become activated, hyper/hyposensitivity of the pathway to physiologic stimulators, altered response kinetics and rewiring of canonical pathways) thus revealing otherwise unseen functional heterogeneity in apparently morphologically and molecularly homogeneous disease groups. When applied to pathways shown to be important in the disease pathology, mapping of signaling networks by SCNP has potential applications in diagnostic testing and drug profiling [39], [42]–[46].

**Table 1:** Examples of nodes and pathways analyzed using SCNP.

Relevant Biology	Modulator	Pathway	Readouts
Growth & Stem Cell Factor Responsiveness	SCF, FLT3L	PI3K, Ras/Raf/Erk	p-Akt, p-Erk, p-S6
Interleukin & Cytokine Responsiveness	IL-27, G-CSF	Jak/Stat	p-Stat1, p-Stat3, p-Stat5
DNA Damage & Apoptosis	Etoposide,	DNA Damage induced Apoptosis	p-Chk2, Cleaved PARP
	Ara-C/Daunorubicin	(Double strand breaks)	
Expression of Surface Markers	n.a.	Drug Transporters &	ABCG2, FLT3, C-Kit
		Growth Factor receptors	

Note: See Table S1 for the complete list of nodes analyzed.

Alterations in kinases, phosphatases and transcription factors can lead to aberrant changes in intracellular protein networks resulting in increased proliferation and survival, and blocks in differentiation, and contributing to the pathogenesis of AML. In this report, using diagnostic non-M3 AML patients, we show that SCNP characterizes AML signaling pathways (Table 1) and provides additional information that is not captured by FLT3 receptor mutational status alone. This is the first step in a series of studies aimed at the development and subsequent validation of functional signaling readouts in the context of FLT3 receptor pathway deregulation; this information provides insight into critical functional dependencies of individual AML on specific signaling pathways, information which is potentially clinically relevant and may ultimately be used to inform selection of patients for treatment with inhibitors of these pathways.

## Results

### Patient and Sample Characteristics

This study utilized two separate cohorts of patients. The first sample set (study one) consisted of 34 cryopreserved diagnostic peripheral blood mononuclear cell (PBMC) samples collected between September 1998 and September 2007 from AML (non-M3) patients treated at hospitals affiliated with the University Health Network (PMH/UHN), University of Toronto. In comparison to the general United States (US) AML population, patients in this group tended to be younger (74%<60 years), female (62%), of Asian ancestry (29%), and had primary refractory (NR) leukemia (74%) (Table 2). Most leukemias were of cytogenetically normal (CN) intermediate-risk cytogenetics (76%) with half of those expressing FLT3-ITD. In study one, sample characteristics of patient age, gender, race and cytogenetic risk were similar in samples negative (FLT3-WT) and positive (FLT3-ITD) for ITD mutations (Table 2). One AML sample had a FLT3 receptor tyrosine kinase domain mutation (TKD). Patient survival curves in the context of presence or absence of FLT3 receptor ITD mutations demonstrated expected trends (Figure S1A) [5]–[8], [10]–[16].

**Table 2:** AML patient clinical characteristics (study 1 and 2).

Donors Are Classified by FLT3-ITD Mutation Status									
		Study 1				Study 2			
		FLT3-ITD Status				FLT3-ITD Status			
Characteristic		ITD	WT	All	P-value^2^	ITD	WT	All	P-value^2^
Age (yr)	N	15	19	34	.	16	67	83	.
	Mean (Std dev)	47.2 (14.34)	52.9 (13.06)	50.4 (13.73)	0.237	51.6 (11.37)	54.0 (12.35)	53.5 (12.14)	0.48
	Median	47.9	52.4	49.1	.	49.7	55.3	55.1	.
	Min, Max	20.7, 71.1	31.0, 74.8	20.7, 74.8	.	34.9, 75.7	25.0, 79.0	25.0, 79.0	.
Age Group	<60 yr	12 (80%)	13 (68%)	25 (74%)	0.697	13 (81%)	50 (75%)	63 (76%)	0.75
	≥60 yr	3 (20%)	6 (32%)	9 (26%)	.	3 (19%)	17 (25%)	20 (24%)	.
Sex	F	9 (60%)	12 (63%)	21 (62%)	1	11 (69%)	35 (52%)	46 (55%)	0.274
	M	6 (40%)	7 (37%)	13 (38%)	.	5 (31%)	32 (48%)	37 (45%)	.
Cytogenetic Groups	Favorable	0 (0%)	1 (5%)	1 (3%)	0.311	0 (0%)	7 (10%)	7 (8%)	0.07
	Intermediate	13 (87%)	13 (68%)	26 (76%)	.	11 (69%)	26 (39%)	37 (45%)	.
	Unfavorable	0 (0%)	3 (16%)	3 (9%)	.	5 (31%)	34 (51%)	39 (47%)	.
	Not Done	2 (13%)	2 (11%)	4 (12%)	.	0 (0%)	0 (0%)	0 (0%)	.
Induction Response^1^	CR	5 (33%)	4 (21%)	9 (26%)	0.462	11 (69%)	44 (66%)	55 (66%)	1
	NR	10 (67%)	15 (79%)	25 (74%)	.	5 (31%)	23 (34%)	28 (34%)	.
Overall Survival (weeks)	Median (95% Conf. Interval)	45.71 (34.47, 56.96)	57.29 (10.11,104.46)	52.14 (35.16, 68.83)	0.040	99.71 (55.78, 143.64)	54.29 (37.49, 71.08)	63.28 (48.49, 78.09)	0.039
CR Duration (weeks)	Median (95% Conf. Interval)	47.00 (30.94, 63.05)	62.86 (30.87, 94.85)	47.00 (30.59, 63.41)	0.875	23.14(−16.20, 62.49)	50.00 (29.44, 70.56)	50.00 (31.34, 68.66)	0.006
Race	Black	0 (0%)	3 (16%)	3 (9%)	.	3 (19%)	3 (4%)	6 (7%)	.
	White	9 (60%)	11 (58%)	20 (59%)	.	7 (44%)	23 (34%)	30 (36%)	.
	Hispanic	0 (0%)	0 (0%)	0 (0%)	.	1 (6%)	4 (6%)	5 (6%)	.
	Asian	6 (40%)	4 (21%)	10 (29%)	0.242	0 (0%)	1 (1%)	1 (1%)	0.238
	Unknown	0 (0%)	1 (5%)	1 (3%)	.	5 (31%)	36 (54%)	41 (49%)	.
FAB	M0	0 (0%)	2 (11%)	2 (6%)	0.234	0 (0%)	1 (1%)	1 (1%)	0.086
	M1	1 (7%)	3 (16%)	4 (12%)	.	5 (31%)	4 (6%)	9 (11%)	.
	M2	5 (33%)	1 (5%)	6 (18%)	.	6 (38%)	27 (40%)	33 (40%)	.
	M4	4 (27%)	4 (21%)	8 (24%)	.	2 (13%)	19 (28%)	21 (25%)	.
	M5	1 (7%)	4 (21%)	5 (15%)	.	3 (19%)	9 (13%)	12 (14%)	.
	Unknown	3 (20%)	4 (21%)	7 (21%)	.	0 (0%)	3 (4%)	3 (4%)	.
	M6	0 (0%)	0 (0%)	0 (0%)	.	0 (0%)	4 (6%)	4 (5%)	.
	Mixed lineage	0 (0%)	1 (5%)	1 (3%)	.	0 (0%)	0 (0%)	0 (0%)	.
	Tri-lineage dysplasia	1 (7%)	0 (0%)	1 (3%)	.	0 (0%)	0 (0%)	0 (0%)	.
FLT3-TKD	Negative	15 (100%)	18 (95%)	33 (97%)	0.367	14 (88%)	63 (94%)	77 (93%)	0.365
	Positive	0 (0%)	1 (5%)	1 (3%)	.	2 (13%)	4 (6%)	6 (7%)	.
Secondary AML	No	15 (100%)	18 (95%)	33 (97%)	0.367	13 (81%)	46 (69%)	59 (71%)	0.318
	Yes	0 (0%)	1 (5%)	1 (3%)	.	3 (19%)	21 (31%)	24 (29%)	.
Chemotherapy	Fludarabine + HDAC	0 (0%)	0 (0%)	0 (0%)	.	0 (0%)	12 (18%)	12 (14%)	0.302
	IA + Zarnestra	0 (0%)	0 (0%)	0 (0%)	.	5 (31%)	21 (31%)	26 (31%)	.
	IDA + HDAC	0 (0%)	0 (0%)	0 (0%)	.	6 (38%)	19 (28%)	25 (30%)	.
	Other	0 (0%)	0 (0%)	0 (0%)	.	5 (31%)	15 (22%)	20 (24%)	.
	Standard 3 + 7	15 (100%)	19 (100%)	34 (100%)	.	0 (0%)	0 (0%)	0 (0%)	.

1 There are 25 primary refractory patients and 6 failed patients in Study 2.

2 P-values were caluclated as follows:

Median survival was estimated from Kaplan-Meier survival curves; the p-value is the log-rank test.

Fisher's Exact test or the standard Chi-Square test was used to compare donors with FLT3-ITD, FLT3-WT, or Unknown status with respect to categorical variables.

The two-sample ttest was used to compare mean ages of FLT3-ITD and FLT3-WT donors; donors with unknown status were not included in the comparison.

The second sample set (study two) consisted of 83 cryopreserved diagnostic bone marrow mononuclear cell (BMMC) samples collected from AML (non-M3) patients treated at MD Anderson Cancer Center (MDACC) between September 1999 and September 2006 (Table 2). This sample set was more representative of the US AML patient population for expected response rates to induction therapy and clinical characteristics except for gender (55% female) and age at diagnosis (76%<60 years). Importantly, FLT3-ITD samples were similar to FLT3-WT samples for patient age, gender, and clinical response to induction chemotherapy (Table 2). Poor risk cytogenetics AML was more frequent in FLT3-WT samples (51%) than in FLT3-ITD samples(31%); FLT3 receptor TKD mutation was present in (2/16) FLT3-ITD and (4/67) FLT3-WT samples. Survival curves in the context of FLT3-ITD showed expected trends (Figure S1B) [5]–[8], [10]–[16].

Ten healthy BMMC taken from male and female volunteers aged between 24–80 years (mean = 66 years) were analyzed as controls. Two BMMC samples were used as controls in study one and study two. Additionally, eight control BMMC samples were analyzed separately in order to characterize FLT3L induced signaling in healthy myeloblasts.

### Comparison of FLT3- WT Signaling in Healthy and AML Samples

**Figure 1:**
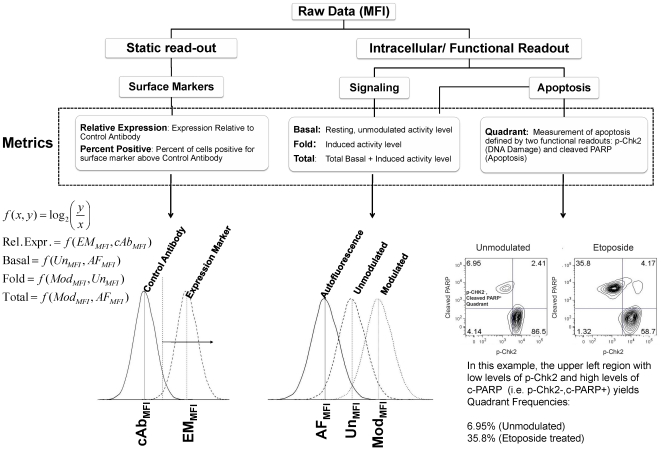
Role of each metric in assessing different aspects of signaling biology. Summary schema of all metrics used in the two studies and the role each has in assessing different aspects of signaling and pathway biology. Median Fluorescence Intensities (MFI) were calculated for leukemic blast cells in each condition and used to compute metrics for Relative Expression of surface expression markers and Basal, Fold and Total measurements of signaling as described in Results. Definitions of metrics used to analyze surface marker expression, signaling data, and apoptosis readouts are shown (i.e. Basal  =  log_2_(Unmodulated_MFI_/Autofluorescence_MFI_).

To analyze modulator-antibody combinations, hereafter defined as signaling nodes, several metrics were developed to describe pathway activity: Basal (basal, modulator-independent activity), Fold Change (modulator-induced), and Total (overall pathway activity) (Figure 1 and Methods). “Node | metric” combinations were used to analyze SCNP data. Study one and two were conducted sequentially. Reagents and cytometers were characterized and calibrated respectively, after the first study, which resulted in some experimental differences between the two studies preventing the merger of raw data. Therefore, the results from each study were analyzed independently. Overall trends are comparable between the studies but absolute values are not. Key pathways analyzed were Ras/Raf/MAPK, PI3K/Akt, PLCγ/CREB, Jak/Stat, and Apoptosis (Table 1) [47]–[51].

**Figure 2:**
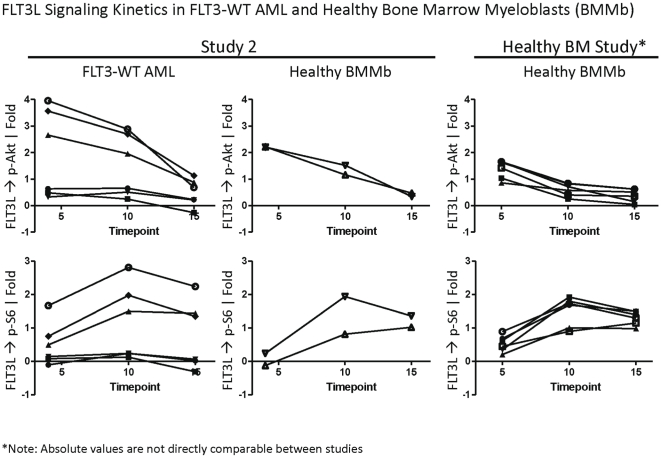
FLT3L Induced Signaling Kinetics in Healthy Bone Marrow Myeloblasts (BMMb) and FLT3-WT AML blasts. Shown are kinetics of FLT3L induced p-Akt (upper) and p-S6 (lower panels) in FLT3-WT AML (left) and healthy control BMMb from Study 2 (center) and additional healthy BMMb samples from a separate cohort (n = 8) (right). While BMMb displayed fairly uniform kinetics of FLT3L induced p-Akt and p-S6, FLT3-WT AML demonstrated a range of kinetic responses including elevated and sustained p-Akt and a rapid, heightened induction of p-S6 compared to healthy BMMb. Additional BMMb were analyzed from a separate study (right panel) to increase the number of healthy controls analyzed. Note: absolute values are not comparable between studies due to different experimental configurations.

**Figure 3:**
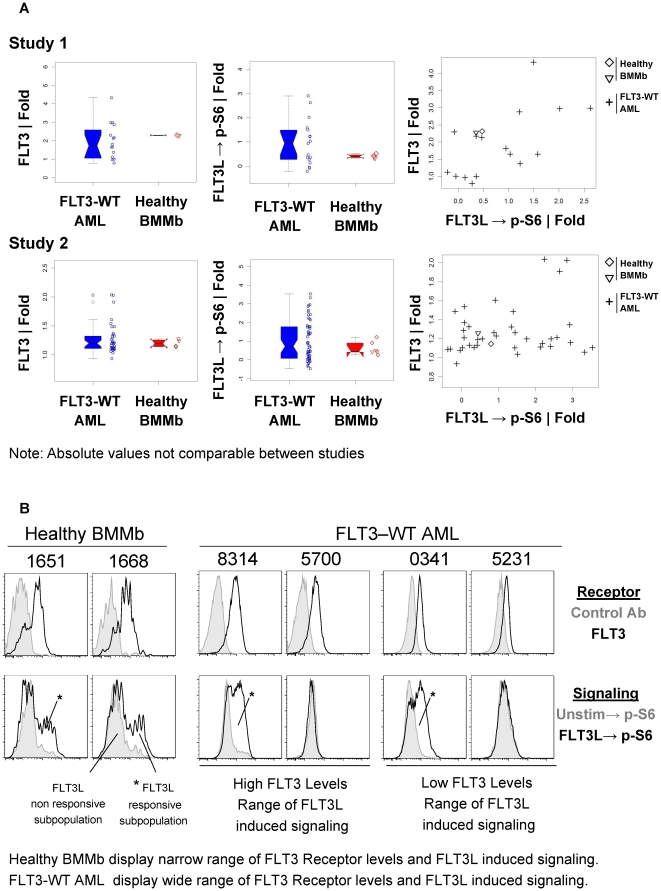
Wider range of FLT3 receptor levels and responsiveness observed in FLT3-WT AML versus healthy BMMb. A) Comparison of FLT3 receptor levels and FLT3L induced signaling (FLT3L→p-S6 |Fold) in FLT3-WT AML samples and healthy BMMb from Study 1 (top panel) and Study 2 (bottom panel). The two distinct healthy BMMb in each study are illustrated by triangle and diamond symbols. Left, Box and whisker plots of FLT3 receptor levels, center: Box and whisker plots of FLT3L induced p-S6, right scatter plot of FLT3 receptor levels and FLT3L induced p-S6. Note: In Box and whisker plots, boxes contain 50% of the sample data with the median values indicated with a horizontal bar. Whiskers contain 1.5× the interquartile range and outliers past this range are shown as individual points. Absolute values are not comparable between studies due to experimental differences in antibody stain time and temperature and choice of α-FLT3 antibody. B) Examples of Healthy BMMb and FLT3-WT AML FLT3 Receptor levels and signaling: Top panel: histograms of FLT3 receptor (black) vs. control antibody (grey); Bottom panel: histograms of basal (grey) or FLT3L induced (black) p-S6. The p-S6 positive FLT3L responsive subpopulations are designated with asterisks. Examples of FLT3-WT AML samples with high FLT3 Receptor levels and low FLT3 Receptor levels are shown.

**Figure 4:**
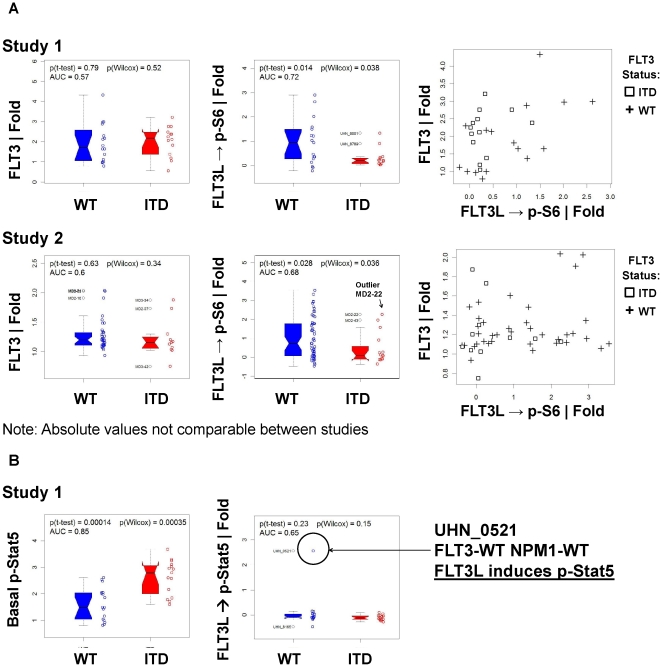
Variable and increased range FLT3L induced PI3K and Raf/Ras/Erk pathways in FLT3-WT versus FLT3-ITD AML. A) Comparison of FLT3 receptor levels and FLT3L induced signaling (FLT3L → p-S6 |Fold) in FLT3-WT (pluses) and FLT3-ITD (squares) AML samples from Study 1 (top panel) and Study 2 (bottom panel). Left, FLT3 receptor levels, center: FLT3L induced p-S6, right combination plot of FLT3 receptor levels and FLT3L induced p-S6. Note: Absolute values are not comparable between studies. B) Example of p-Stat5 | Basal and FLT3L → p-Stat5 | Fold levels from Study 1 demonstrates a FLT3L → p-Stat5 response from a FLT3-WT NPM1-WT sample (UHN_0521).

SCNP was utilized to compare WT FLT3 receptor signaling in healthy BMMb versus FLT3-WT leukemic blasts (see Methods for gating hierarchy). As expected based on data from leukemic cell line experiments, FLT3L activated the MAPK and PI3K pathways inducing increased levels of p-Akt and p-S6 [30]–[37]. However, there were kinetic differences for WT FLT3 receptor signaling in healthy BMMb versus AML blasts. While increases in p-Erk and p-Akt in response to FLT3L were observed at earlier time points (Figure 2), activation of these proteins was largely diminished by 15′ in healthy BMMb likely due to regulatory feedback mechanisms [52]. In contrast, sustained p-Erk and p-Akt was observed in a number of FLT3-WT AML samples at 15′ compared to healthy BMMb (Table 3) and elevated signaling was evident at earlier timepoints (Figure 2). In healthy myeloid cells, FLT3L induced a narrow range of signaling with no increases in p-Stat5 (Table S2A, Table 3). In contrast, in FLT3-WT AML samples FLT3L induced a wide range of signaling independent of the level of FLT3 receptor [although samples with the lowest FLT3 levels demonstrated no FLT3L induced signaling (Table 3, Figure 3A, 3B, 4A)]. As compared with healthy BMMb, FLT3-WT samples displayed deregulated FLT3 receptor signaling ranging from having no induced signaling, to having sustained levels of p-CREB, p-Akt, p-Erk at 15′, earlier induction of p-S6, and in one case a 2.57 fold increase in p-Stat5, after modulation with FLT3L (Table 3, Figure 2). In agreement, standard deviations from measures of FLT3 signaling were higher in FLT3-WT AML than in healthy BMMb. In addition, the variance in FLT3 receptor signaling was statistically different (p-value  =  0.003, Levene's test) between the FLT3-WT AML and healthy BMMb samples (Table S2A) [53]. Furthermore, although individual healthy and AML samples displayed uniform FLT3 receptor staining, FLT3L responsiveness and induction of p-S6 was only observed in a fraction of cells (Figure 3B).

**Table 3:** Summary table comparing FLT3 Receptor and FLT3L induced signaling between normal BM Myeloblasts (BMMb) and FLT3-WT AML.

	Study 1				Study 2			
	Healthy BMMb	FLT3-WT AML			Healthy BMMb	FLT3-WT AML		
Node | Metric:	Ave	Ave	Min	Max	Ave	Ave	Min	Max
Surface Markers:								
FLT3 Receptor | Relative Expression	2.30	1.97	0.79	4.33	1.20	1.27	0.94	2.04
FLT3L induced signaling:*								
FLT3L → p-S6	0.51	0.96	−0.22	2.91	0.62	1.03	−0.47	3.54
FLT3L → p-Akt	0.50	0.63	−0.13	2.31	0.43	0.50	−0.47	1.66
FLT3L → p-Erk	0.19	0.33	−0.18	0.98	0.41	0.25	−0.27	0.95
FLT3L → p-CREB	−0.11	0.37	−0.20	1.29	0.18	0.12	−0.17	0.40
FLT3L → p-Stat5	−0.06	0.08	−0.47	2.57	0.41	0.35	−0.01	0.55
FLT3L → p-PLCγ2	−0.21	−0.11	−0.53	0.21	0.01	0.04	−0.04	0.09

* Signaling assesed at 15′.

Note: Mean values are not directly comparable between Study 1 and Study 2 due to experimental configuration and methodological improvements between studies.

No statistics were applied for the Healthy BMMb comparison within Study1 or Study2 alone due to the low number of healthy donors.

See Table S2A for a statistical analysis of varaince for FLT3L induced signaling in FLT3-WT AML and Healthy BMMb samples.

### Signaling and Apoptosis Nodes Stratify FLT3-WT and FLT3-ITD AML Samples

To assess signaling differences between FLT3-WT and FLT3-ITD AML samples, we used SCNP to measure the activities of pathways that regulate hematopoietic cell proliferation and differentiation, drug transport, apoptosis, and DNA damage response/apoptosis (Table 1, Table S1).

**Figure 5:**
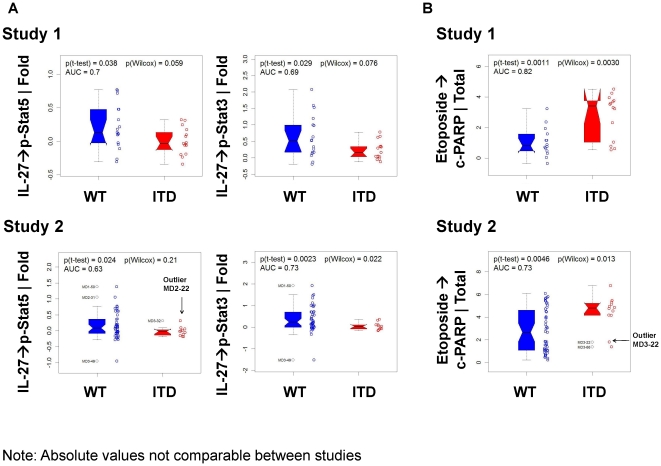
Wider range of modulation in Jak/Stat and Apoptosis pathways in FLT3-WT versus FLT3-ITD AML. A) IL-27 → p-Stat5, p-Stat3 | Fold signaling responses for FLT3-WT and FLT3-ITD samples from Study 1 (top row) and Study 2 (bottom row) were greater and more variable in FLT3-WT compared to FLT3-ITD AML samples. B) Etoposide → cleaved PARP | Total levels for FLT3-WT and FLT3-ITD samples from Study 1 (top row) and Study 2 (bottom row) demonstrate increased *in vitro* apoptosis after continuous etoposide treatment in FLT3-ITD vs. FLT3-WT samples. Note: Absolute values are not comparable between studies.

**Figure 6:**
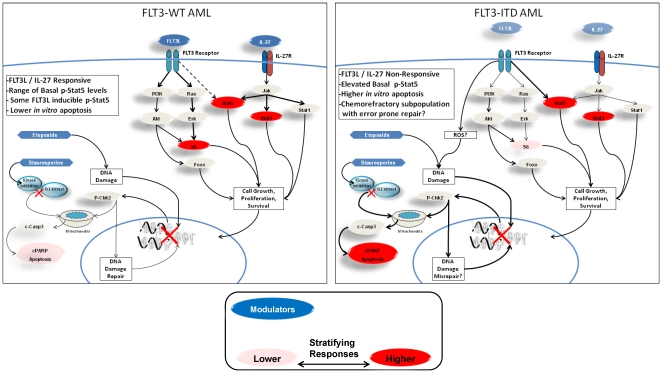
Overview of pathway differences observed between FLT3-ITD and FLT3-WT AML samples across the two studies. Illustration of pathway differences between FLT3-ITD and FLT3-WT samples. Red coloring of readouts and bold lines indicate increased pathway activity. White coloring and non-bold lines indicate decreased pathway activity. Dotted line from FLT3L to p-Stat5 in FLT3-WT samples indicates connectivity in a subset of patients. Red “X” indicates DNA Damage. Faded modulators (FLT3L, IL-27) in FLT3-ITD indicate decreased modulator induced signaling.

Univariate analysis, unadjusted for multiple testing, was performed sequentially and independently on the two study cohorts to identify signaling nodes commonly associated with FLT3 receptor mutational status (Table 4). Seventy seven of 304 and 48 of 201 node/metrics tested in studies one and two respectively distinguished FLT3-ITD from FLT3-WT AML patient samples with an AUC of ROC ≥0.6 and *p*≤0.05 (Tables S3, S4). Analysis of false discovery rate for both studies showed this frequency to be significantly greater than the number of signaling nodes that would be expected to be significantly different between the two groups just by chance (p  =  0.0009 study one and p<0.0001 study two respectively) (Figure S2). Stratifying nodes that distinguished FLT3-ITD from FLT3-WT samples in both studies represented distinct biological networks including Jak/Stat, PI3K and apoptosis pathway readouts (Figures 4, 5, 6, Table 4).

**Table 4:** Summary table of common stratifying pathways between FLT3-WT and FLT3-ITD signaling in AML samples in both studies.

			Study 1						Study 2					
Node | Metric:	FLT3-WT AML	FLT3-ITD AML	AUC_ROC_	t-test *P*	Wilcox. *P*	Mean Value of WT/ITD			AUC_ROC_	t-test *P*	Wilcox. *P*	Mean Value of WT/ITD		
PI3K/S6 induced Signaling:														
FLT3L → p-S6 | Fold	**↑**		0.72	0.014	0.038	0.96	/	0.30	0.68	0.028	0.036	1.03	/	0.43
FLT3L → p-S6 | Total	**↑**		0.80	0.003	0.003	1.46	/	0.45	0.65	0.035	0.119	4.09	/	3.27
Jak/Stat Signaling:														
IL-27 → p-Stat3 | Fold	**↑**		0.69	0.029	0.076	0.64	/	0.23	0.73	0.002	0.022	0.37	/	0.04
IL-27 → p-Stat5 | Fold	**↑**		0.70	0.038	0.059	0.21	/	0.00	0.63	0.024	0.207	0.15	/	−0.02
Functional Apoptosis Response:														
Etoposide → c-PARP | Total		**↑**	0.82	0.001	0.003	1.06	/	2.79	0.73	0.005	0.013	2.81	/	4.45

**↑  =  higher signaling response in indicated molecular group.**

For detailed information for each Study see Tables S3 and S4.

Note: Mean values are not directly comparable between Study 1 and Study 2 due to experimental configuration and methodological improvements between studies.

### FLT3 Receptor Levels and Signaling Activity in FLT3-WT and FLT3-ITD Samples

Although FLT3-ITD and FLT3-WT samples expressed similar levels of the FLT3 receptor, FLT3L induced distinct signaling responses in the two types of sample sets (Figures 4A, B). In addition, basal levels of p-Erk, p-Akt, and p-S6 did not differ significantly between FLT3-ITD and FLT3-WT blasts (Figure S3A). After modulation with FLT3L, FLT3-ITD samples showed lower levels of induced PI3K and MAPK pathway activation compared to FLT3-WT samples (Figure 4, Tables S3, S4, Figure S4). This was particularly evident for FLT3L induction of p-S6 (Figure 4A) which, in both studies, by univariate analysis, discriminated between FLT3-ITD and FLT3-WT samples (FLT3L→p-S6 | Fold, AUC of ROC 0.72 and 0.68 for study one and study two respectively). In both studies, a range of basal p-Stat5 levels was observed in FLT3-ITD and FLT3-WT samples, with higher levels observed in FLT3-ITD samples (Figure 4B). Notably, while FLT3L stimulation did not further increase p-Stat5 in FLT3-ITD samples, FLT3L was able to increase p-Stat5 in one FLT3-WT (and NPM1-WT) sample (Figure 4B), a response not observed in healthy myeloid cells, suggesting deregulated FLT3 receptor signaling both in the presence and absence of known FLT3 receptor mutational alterations. Notably, for most FLT3L inducible readouts a wider range of responses was observed in FLT3-WT samples while FLT3-ITD samples behaved more uniformly (Figure 4, Figure S4). In agreement, FLT3-WT AML demonstrated higher standard deviations of FLT3 induced p-S6 than FLT3-ITD AML, and the difference in variance between the groups was statistically significant (p-value<0.001, Levene's test) (Table S2A) [53].

### Distinct Jak/Stat Signaling in FLT3-WT and FLT3-ITD Samples

While a wide range of IL-27 →p-Stat responses were observed in the FLT3-WT samples, FLT3-ITD samples behaved much more uniformly and displayed minimal responsiveness to IL-27 stimulation. A comparison of basal p-Stats levels for FLT3-ITD versus FLT3-WT showed that p-Stat1, p-Stat3, and p-Stat5 were increased in FLT3-ITD samples in study one but not in study two (Figure S3B). Furthermore, IL-27 mediated Jak/Stat pathway activity was lower in FLT3-ITD samples compared to FLT3-WT samples with significantly lower induction of p-Stat3 (p≤0.029) and p-Stat5 (p≤0.038) in both studies (Figure 5A). The IL-27 →p-Stat 3 | Fold signaling node in univariate analysis stratified FLT3-ITD positive from FLT3-ITD negative samples (AUC 0.69 study one and AUC 0.73 study two respectively) (Table S3, S4).

### Distinct Apoptosis Responses seen in FLT3-WT and FLT3-ITD Samples

In order to determine the integrity of the DNA damage response (DDR) and apoptotic machinery, the ability of etoposide to induce DNA damage (increased p-Chk2 levels) and apoptosis (increased cleaved PARP levels), was measured in leukemic blasts. FLT3-ITD samples were more sensitive to *in vitro* apoptosis than FLT3-WT samples (etoposide → c-PARP| Total AUC 0.82 study one and AUC 0.73 study two, respectively) (Figure 5B, Tables S3, S4). Similar results were observed in both studies using other mechanistically distinct apoptosis-inducing agents such as staurosporine, a pan kinase inhibitor, and in study two, Ara-C/Daunorubicin (Tables S3, S4)

### Correlation between Stratifying Nodes

**Figure 7:**
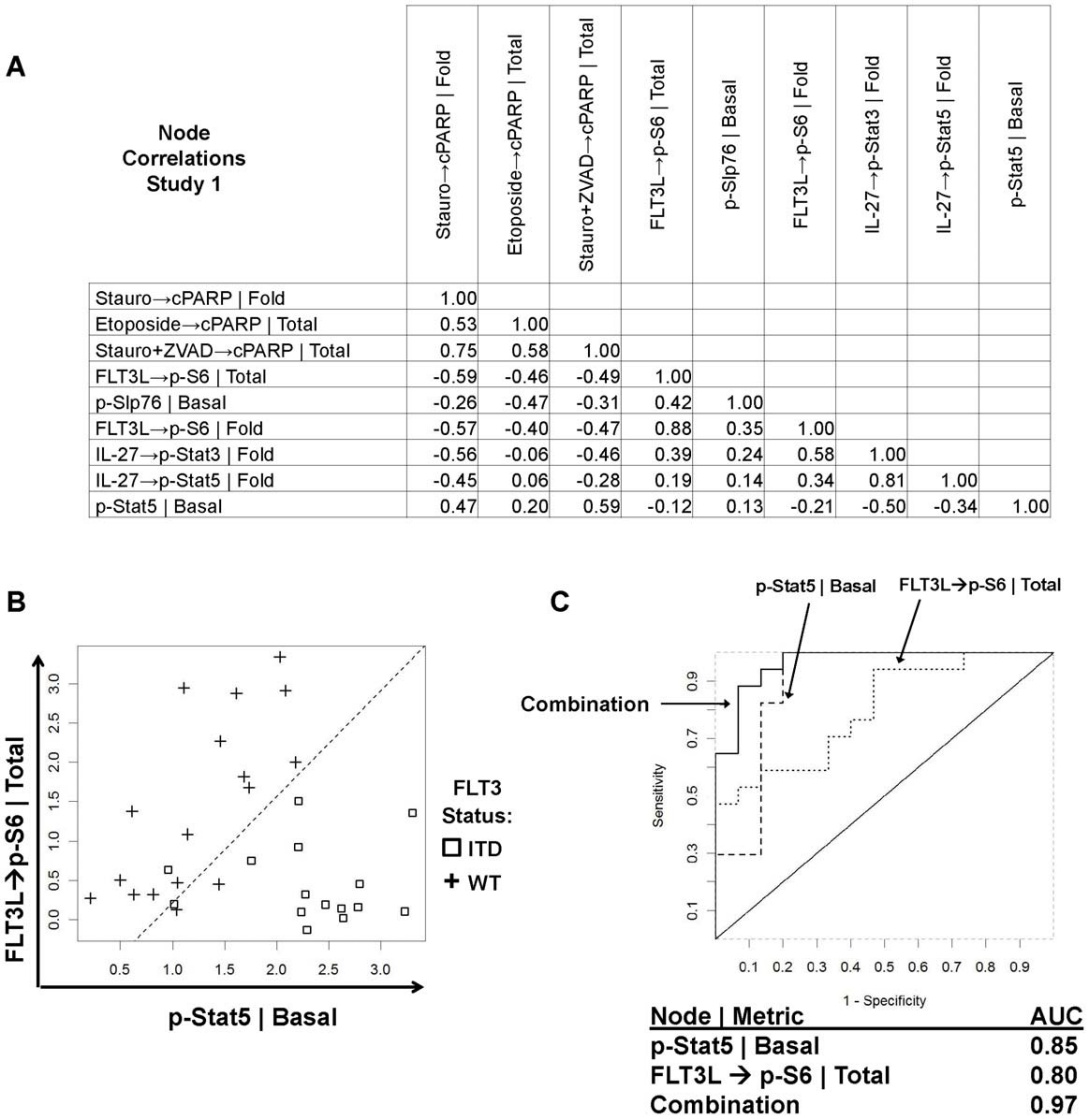
Correlations and multivariable analysis of common nodes which stratify FLT3-WT and FLT3-ITD AML samples in Study 1. A) Correlation (R) Values between FLT3-WT vs. FLT3-ITD stratifying nodes demonstrate correlated nodes/pathways (IL-27→p-Stat3 | Fold, IL-27 →p-Stat5, | Fold: R = .81) and non-correlated nodes/pathways (IL-27 → p-Stat5, | Fold, FLT3L → p-S6 | Total: R = .19) suggesting that distinct pathways may provide independent information in characterizing the biology of FLT3-ITD and FLT3-WT AML samples. An R value of 0 indicates no correlation, positive R values (0–1) indicate positive correlation and negative R values (−1 to 0) indicate negative correlation. Correlations based on 25 donors with data for all nodes. See Table S5 for additional nodes. B) Multivariate analysis showing combining pairs of nodes can improve stratification over single nodes for stratifying FLT3-ITD and FLT3-WT samples. Shown is a combination plot of p-Stat5 | Basal, FLT3L → p-S6 | Total with FLT3-ITD samples. The dotted line shows how stratification between FLT3-WT (pluses) and FLT3-ITD (squares) is improved using combinations of independent stratifying nodes. C) AUC_ROC_ values for individual nodes (p-Stat5 | Basal, FLT3L → p-S6 | Total), and the nodes in combination.

Although the signaling nodes were analyzed independently in the primary analysis, several of the top-ranking nodes stratifying FLT3-ITD from FLT3-WT AML samples were correlated with each other. Pearson correlation coefficients computed for all signaling nodes with a p≤0.05 from study one (Table S5) showed (as it would be expected) correlation between nodes measuring signaling events in the same pathway. For example, the following node/metrics: IL-27→p-Stat3 | Fold and IL-27→p-Stat5 | Fold (R  =  0.81) or Thapsigargin→p-CREB | Fold and PMA→p-CREB | Fold (R  =  0.87), suggest common signaling pathways between the nodes in these pairs. By contrast, poor correlations were observed between nodes measuring signaling events in different pathways (such as Thapsigargin→p-CREB | Fold and IL-27→p-Stat5 | Fold (R = 0.04), suggesting that these nodes are measuring distinct signaling pathways and might be combined to produce a multivariate model for prediction of FLT3 receptor mutational status with higher predictive value (Figure 7).

### Association between Multiple Signaling Nodes and FLT3-ITD Status –Multivariate Analysis using Linear Regression

To determine if different signaling nodes in combination could provide superior stratification of FLT3-ITD and FLT3-WT AML samples, all possible pairs of the 80 signaling nodes with AUC of the ROC ≧0.7 or lowest p≤0.05 from study 1 (Table S3) were evaluated for their ability to improve stratification of the FLT3 receptor mutational status (Table S6). Given the limited size of this data set, this modeling exercise was performed to explore potential combinations within or across pathways that might form the basis of future studies. Of note, all combinations that had an AUC greater than the best single node/metric within the combination were assessed (please see Materials and Methods). The AUC for the models ranged from 0.89 to 0.99 (Table S6). As expected, the probability of two nodes to complement one another was higher if the nodes participated in different signal transduction pathways: e.g. combining the nodes p-Stat5 | Basal (AUC = 0.85) and FLT3L→p-S6 | Total (AUC = 0.80), (R  =  −0.12), yields an improved AUC of 0.97 (Figure 7A, 7B, 7C).

### FLT3L and IL-27 Induced Signaling in FLT3-ITD, NPM1 Molecular Subgroups

To understand how FLT3 receptor and NPM1 mutational status relate to intracellular biological pathways in diagnostic AML samples, we assessed IL-27 induced Jak/Stat signaling and FLT3L induced PI3K and Raf/Ras/MAPK signaling responses in FLT3 receptor and NPM1 molecular defined subgroups. NPM1 mutational status was available for all patients in study one but only for a subset in study two; hence the interpretation of the results of these analysis are limited by the small number of samples in each subset. For all nodes analyzed, the FLT3-WT/NPM-WT subgroup demonstrated the most variable signaling responses and often contained samples with the most elevated signaling (Figure S5). In contrast, within FLT3-ITD/NPM1 mutated patients, IL-27-induced and FLT3L-induced signaling appeared more uniform and generally lower compared to FLT3-WT/NPM-WT samples. FLT3-WT/NPM1-WT samples demonstrated the highest variance among FLT3 NPM1 subgroups for IL-27 and FLT3L signaling and demonstrated significantly higher variance compared to both FLT3-ITD subgroups (Table S2B). Of note, the largest differences in variance were observed between FLT3-WT/NPM-WT and FLT3-ITD/NPM-Mutated samples (Table S2B).

### Clinical Implications of These Studies

To assess if functional intracellular pathways characterization using SCNP may stratify (beyond molecular characterization) AML for clinical outcomes, the signaling profiles of samples with clinical outcomes not predicted by molecular findings (here called “outliers”) were analyzed.

Signaling profiles were assessed in samples from two groups of Cytogenetically Normal (CN) AML patients, each representing a clinically extreme example of an “outlier” (based on the molecular characterization): 1) FLT3-WT AML who experienced disease relapse within three months after initial remission and 2) FLT3-ITD AML in complete continuous disease remission for two or more years. In the first study there were no patient samples that met these two criteria. In the second study there were two FLT3-WT and two FLT3-ITD samples respectively (Table 5). Clinical characteristics of CN AML samples used for this subset analysis from both studies are presented in Table S7.

**Table 5:** Identification of cytogenetically normal clinical outliers.

	FLT3 Status	Relapse <3 Months	Remission >2 Years
**Study 1:**	**ITD**	0	0
	**WT**	0	0
**Study 2:**	**ITD**	2	**2**
	**WT**	**2**	9

**Figure 8:**
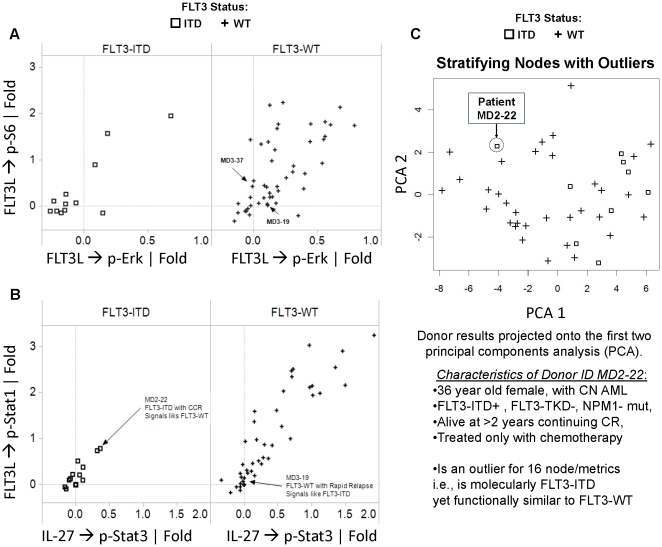
Signaling profiles of “clinical outliers”. A) Scatter plot of FLT3L → p-Erk | Fold (X-axis) and FLT3L → p-S6 | Fold (Y-axis) for FLT3-ITD samples (left, squares) and FLT3-WT samples (right, pluses). Samples MD3–19 and MD3–37, FLT3-WT samples with Rapid Relapse, display low FLT3L induced signaling, similar to FLT3-ITD samples, and are shown. B) Scatter plot of IL-27 → p-Stat3 | Fold (X-axis) and IL-27 → p-Stat1 | Fold (Y-axis) for FLT3-ITD samples (left, squares) and FLT3-WT samples (right, pluses). Sample MD3–19 (FLT3-WT rapid relapse sample) displays low IL-27 induced signaling, similar to FLT3-ITD samples, and is shown. Also indicated is FLT3-ITD outlier MD2–22 which demonstrates higher IL-27 induced signaling similar to FLT3-WT samples. C) Principal Component Analysis of stratifying nodes with outliers illustrates that while most FLT3-ITD samples cluster together (squares), FLT3-WT samples (pluses) are more heterogeneous and sample MD2–22 (circled) signals distinctly from other FLT3-ITD samples. This indicates that clinical outlier MD2–22, a FLT3-ITD sample with remission >2 years, signals in many nodes like a FLT3-WT sample.

The wide range of signaling responses observed in FLT3-WT AML samples made identification of signaling outliers in this subgroup challenging. Examination of the signaling profiles in the two FLT3-WT samples with relapse <3 months (MD3–19 and MD3–37) showed low p-S6 and p-Erk in response to FLT3L, similar to induced signaling observed in FLT3-ITD samples (Figure 8A). In addition, MD3–19 showed minimal IL-27 mediated Stat phosphorylation, similar to FLT3-ITD samples (Figure 8B), suggesting that these FLT3-WT samples from patients with rapid disease relapse might share similar biology with FLT3-ITD samples in certain pathways. In contrast, identification of FLT3-ITD signaling outliers was aided by the narrow range of signaling responses observed in this sample set. In the CN FLT3-ITD sample group, two patients remained in complete continuous remission for two or more years, one (MD2–22) having been treated with chemotherapy alone and the other (MD3–22) treated with an allogeneic stem cell transplant (as per NCCN guidelines). Since MD3–22 received high intensity post-remission therapy we focused on signaling associated with sample MD2–22 obtained from a patient who received high dose cytarabine similar to what is recommended for “low risk” cytogenetic leukemia. We found that the FLT3-ITD MD2–22 sample signaling profile was closer to FLT3-WT as illustrated by the first two principal components of PCA Analysis using FLT3 stratifying nodes (Figure 8C). This observation was further reinforced by the number of nodes (16) for which MD2–22 was an outlier among the FLT3-ITD group (i.e. outside of 1.5 times the inter-quartile from the median for FLT3-ITD as shown in Table S8). These nodes included those from the Jak/Stat pathway PI3K and MAPK pathways (Figure 5A). A following molecular analysis of this sample indicated the presence of an NPM1 gene mutation although this information was not available at the time of post-remission treatment.

We next correlated SCNP signaling and apoptosis nodes with remission duration in AML samples in the context of FLT3 molecular status. This study was limited to FLT3-WT AML samples due to insufficient FLT3-ITD samples with CR duration greater than two years. Within FLT3-WT AML samples, we found that higher measures of induced apoptosis (i.e. Ara-C/Dauno→C-PARP | Fold) were associated with CR duration greater than two years (AUC_ROC_: 0.92) (Figure S6).

## Discussion

While genetic and molecular heterogeneity may ultimately underlie the different responses to therapy observed among patients with AML, there appears to be a finite number of intracellular signaling pathways through which these differences are expressed. These pathways (which include PI3K/Akt, Ras/Raf/MAPK, and Jak/Stat) play important roles in proliferation, survival, cell cycle progression, and differentiation in both normal and leukemic hematopoietic cells [54].

The present studies illustrate the value of using SCNP to characterize the biologic complexity of AML at the signaling pathway level. Although this approach has been applied previously to AML using a limited panel of nodes [39], this study describes the high-throughput application of the technology, using a standardized 96-well format, examining a broader panel of nodes (including multiple previously unexamined biological pathways in the context of FLT3-ITD status) in a larger numbers of AML patients and in independently assayed sample sets. Results from this study show the existence of significant differences in modulated signaling networks between healthy bone marrow myeloblasts and leukemic AML blasts and, in the latter group, between FLT3 receptor molecularly defined subsets.

While much has been learned from experiments in leukemic cell lines there is little published data on WT FLT3 receptor signaling in healthy bone marrow myeloblasts (BMMb) and primary leukemic blasts [2], [55]. The broad functional assessment of biologically relevant signaling pathways in AML blasts and BMMb conducted in this study shows a spectrum of signal transduction deregulation not previously described in primary AML samples. In fact, the current investigation represents the first analysis comparing basal and modulator-induced activity of multiple distinct pathways such as Jak/Stat, PI3-kinase/Akt/S6 and the Ras/Raf/Erk/S6, phosphatase/reactive oxygen species, and DDR/apoptosis pathways in the context of FLT3 status. To our knowledge this is also the first study that measured FLT3 receptor expression levels in conjunction with signaling profiles in primary leukemia samples. Although FLT3 receptor levels were similar between the FTL3-WT and FLT3-ITD AML groups, FLT3-ITD expressing samples displayed attenuated responses to FLT3L, as measured by induced levels of p-S6, p-Erk, p-Akt and p-CREB versus their FLT3-WT counterparts (Tables 4, S3, S4 and Figure S4). FLT3-ITD expressing cell lines have been reported to have ligand independent signaling with increased levels of basal p-Erk and p-Akt [38]. However, in the primary leukemic blasts tested in this study, basal levels of p-Erk, p-Akt, and p-S6 did not differ significantly between FLT3-ITD and FLT3-WT blasts (Figure S3A). Of note, most studies examining FLT3-ITD in cell lines, have introduced the FLT3-ITD mutation into an already transformed cell with existing mutations and alterations. Overall these data suggest the greater dependence of FLT3L inducibility of these signaling networks in FLT3-WT AML and demonstrate FLT3L-independence in FLT3-ITD samples.

Canonical Stat pathways, activated by cytokines, regulate an array of cellular functions ranging from survival and apoptosis to proliferation and differentiation [56]. In this study there was no basal or FLT3L-induced activation of p-Stat5 in healthy marrow myeloblasts while AML marrow blasts showed a wide range of basal p-Stat5 expression even in the absence of FLT3-ITD. Prior studies have shown that basal p-Stat5 levels are constitutively high in cell lines that have been transfected with FLT3-ITD genes [32]–[36], [38], [57] and recently increased expression of p-Stat5 in AML blasts was found to be highly predictive of the presence of FLT3-ITD mutations [41]. Consistent with these studies we found FLT3-ITD samples expressed increased basal levels of p-Stat1, p-Stat3 and p-Stat5 compared to FLT3-WT samples in study one and in both studies FLT3-ITD AML samples displayed a uniformly limited range in basal p-Stat5 levels compared to FLT3-WT samples. Additionally, in contrast to healthy bone marrow myeloblasts, FLT3L induced p-Stat5 in some FLT3-WT samples, demonstrating deregulated FLT3 receptor signaling even in the absence of FLT3 mutational alterations.

This study is also the first to show different signaling responses between FLT3-WT and FLT3-ITD in AML samples for IL-27 induced Jak/Stat pathway activity. Most studies characterizing the biology of IL-27 have been performed on lymphocytes where this cytokine plays a major role in immune regulation. However, the IL-27 receptor is present on other cell types, including those of the myeloid lineage, where its activation has been shown to enhance proliferation and differentiation of mouse and human hematopoietic stem/progenitor cells [50], [58]. In study one, increased levels of basal p-Stat1 and p-Stat5 were observed for FLT3-ITD compared to FLT3-WT samples. Our data suggest these FLT3-ITD samples are less responsive to IL-27 mediated Stat signaling, likely because they already display elevated Stat pathway activity. This cytokine independence could contribute to the poor clinical outcome observed within FLT3-ITD patients.

Finally, analysis of the apoptosis pathway showed that FLT3-ITD samples were more sensitive in vitro to apoptosis inducing agents than FLT3-WT samples. Aside from differences in cell source (primary leukemia blasts versus cell lines) the continuous exposure of cells to DNA damaging drugs may account for the difference between our data and published literature, where the introduction of FLT3-ITD into cell lines has been associated with resistance to DNA damage induced apoptosis [36], [38]. Recent studies suggest that FLT3-ITD AML samples are more sensitive to long term drug exposure due to deficient S-phase checkpoints as compared to FLT3-WT samples [59]. Future studies that examine longitudinal samples pre- and post-therapy could be informative in defining the basis of the secondary chemo-resistance characteristic of FLT3-ITD samples. In addition, while these results may seem somewhat counterintuitive to the clinical findings that patients with FLT3 ITD leukemia experience worse overall survival and shorter disease-free remission, to date the presence of FLT3-ITD has not been associated with response to induction therapy [17].

Taken together these data show that SCNP uncovers important heterogeneity in AML and has potential as a platform for understanding leukemia pathway dependency in the individual patient, information that could be valuable for the selection of therapeutic strategies in the era of personalized medicine. This method promises to reduce the complicated genetic picture that is emerging for AML to the functional status of a few distinct signaling pathways.

However, whether the SCNP methodology will provide clinically useful and actionable information beyond existing molecular markers remains to be proven (in this study no data were available on additional molecular alterations).

Lastly, multiple therapeutics that target FLT3 receptor (e.g., CEP701, PKC412, AB220) are in development for the treatment of AML. To date, the characterization of AML based on the mutational status of the *FLT3* gene has shown not to be very informative in predicting the activity of any of these FLT3 receptor inhibitors and their effects on signaling transduction remains unknown. In this regard, SCNP could be tested as a tool to identify AML patients who could benefit from administration of such inhibitors alone or in combinations with other standard agents and/or targeted inhibitors. Further studies in the context of clinical trials are warranted.

## Materials and Methods

### Ethics Statement

In accordance with the Declaration of Helsinki, all patients provided written informed consent for the collection and use of their samples for research purposes. All studies were approved by Institutional Review Boards (IRB): University Health Network Research Ethics Board (study one), M.D. Anderson Cancer Center Institutional Review Board (study two), and Independent Review Consulting, Inc. (Healthy BMMC Study). Clinical data were de-identified in compliance with Health Insurance Portability and Accountability Act regulations.

### Patient Samples

Sample inclusion criteria included diagnosis of AML, French-American-British classification as M0 through M7 AML (excluding M3), collection prior to the initiation of induction chemotherapy, molecular determination of the presence or absence of FLT3-ITD, post-thaw cell viability of >50% and >500 cells in the leukemic cell population (defined below). All samples underwent fractionation over Ficoll-Hypaque prior to cryopreservation with fetal calf serum and 10% dimethyl sulfoxide and storage in liquid nitrogen.

The first sample set consisted of 34 cryopreserved peripheral blood mononuclear cell (PBMC) samples collected from AML (non-M3) patients treated at hospitals affiliated with the PMH/UHN, University of Toronto, between September 1998 and September 2007. Induction chemotherapy consisted of one cycle of standard cytarabine-based induction therapy (daunorubicin 60 mg/m^2^ × 3 days, cytarabine 100–200 mg/m^2^ continuous infusion × 7 days). The second sample set consisted of 83 cryopreserved BMMC samples collected from AML (non-M3) patients treated at MDACC between September 1999 and September 2006. Induction chemotherapy consisted of one or two cycles of cytarabine (200 mg/m^2^ to 3 g/m^2^) in combination with an anthracycline (daunorubicin or idarubicin) or an additional anti-metabolite (e.g., fludarabine or troxacitabine), and sometimes an experimental agent. However, due to differences in cell number post-thaw in this study, the number of patients for which data was available for a given node/metric varied between 83 and 9. Standard clinical and laboratory criteria were used for defining complete response (CR) in both studies [60].

A total of ten healthy BMMC were analyzed in these studies. Two healthy BMMC controls (obtained from All Cells) as well as 2 cell lines were used in studies one and two as assay controls. Additionally, eight healthy BMMC controls were obtained from Williamson Medical Center and assayed in a separate study.

### Analysis of FLT3 Receptor Mutational Status

In study one, an aliquot of cells were taken from each sample and DNA and RNA were isolated using TRIzol Reagent (Invitrogen) as described by the manufacturer. In study two, *FLT3 receptor* gene mutational status was assessed at MDACC.

For the analysis of internal tandem duplication of the FLT3 receptor gene, PCR was performed on 500 ng genomic DNA using published primers 11F and 12R as described to identify ITD insertions in the JM and TK1 domains [14] using AmpliTaq Gold DNA polymerase (Applied Biosystems) and 3% agarose gels. For samples with no available genomic DNA, the PCR assay was performed with 100 ng of cDNA prepared using the High Capacity RNA-to cDNA kit (Applied Biosystems).

For the screening of FLT3 receptor D835 mutation by RFLP analysis PCR was performed on 500 ng genomic DNA using published primers 17F and 17R as described [61]. 5 µl of PCR product was digested with 5 U of EcoRV for 1.5 hours at 37°C then analyzed by gel electrophoresis. For samples with no available genomic DNA, the D835 PCR assay was performed using 100 ng of cDNA and reverse primer FLT3-D835R2, 5′-TTGCCCCTGACAACATAGTTGGA-3′, designed for a cDNA template.

### Analysis of NPM1 Mutational Status

In study one, Real-time quantitative PCR assays for NPM1 mutations A and B: RQ-PCR were performed on 100 ng cDNA using published primers and Taqman probe cNPM-F, c. Probe, cNPM mut.A-R, cNPM mut.B-R as described [62] except 40 PCR cycles were used. In study two, standard PCR assays were performed for detection of NPM1 mutations with forward primer 5′-[6-FAM]-GATGTCTATGAAGTGTTGTGGTTC-3′ and reverse primer 5′-GTTTCTAAGGACAGCCAGATATC.

### SCNP Assay

The analysis included response to chemokines, cytokines, growth factors such as: SCF and FLT3L- mediated PI3K/Akt and MAPK pathway activation (important for maintaining the hematopoietic stem cell pool [47], [48]; G-CSF- mediated Jak/Stat pathway activation (important for neutrophilic differentiation of hematopoietic progenitor cells [49]; and interleukin (IL)-6 family members, including IL-27, mediated Jak/Stat pathway activation (important in regulating proliferation and differentiation of hematopoietic stem cells [50]. Drug transporter expression levels, known to be associated with adverse prognosis in AML, [63], [64] and surface myeloid growth factor receptors levels such as c-Kit and FLT3 receptors, were also measured. DNA Damage Response (DDR) and apoptosis pathways were measured using p-Chk2 and cleaved PARP after *in vitro* exposure of AML samples to etoposide, Ara-C/daunorubicin or staurosporine.

SCNP assays were performed as described previously [39]. Cryopreserved samples were thawed at 37°C, washed, and centrifuged in PBS, 10% FBS, and 2 mM EDTA. The cells were re-suspended, filtered to remove debris, and washed in RPMI cell culture media 1% FBS, before staining with Aqua Viability Dye to distinguish non-viable cells. The cells were re-suspended in RPMI, 1% FBS, aliquoted to 100,000 cells/condition, and rested for 1–2 hours at 37°C. For apoptosis assays, cells were incubated for 6h with Staurosporine or 24 hours with cytotoxic drugs (e.g., etoposide or Ara-C and daunorubicin) and re-stained with Aqua Viability Dye. For all other assays, cells were incubated with modulators (Table S1) at 37°C for 3–15 minutes. After exposure to modulators, cells were fixed with 1.6% paraformaldehyde (final concentration) for 10 minutes at 37°C, pelleted and permeabilized with 100% ice-cold methanol, and stored at −80°C. Subsequently, cells were washed with FACS buffer (PBS, 0.5% BSA, 0.05% NaN_3_), pelleted, and stained with cocktails of fluorochrome –conjugated antibodies (Table S9). These cocktails included antibodies against 2 to 5 phenotypic markers for cell population gating (e.g., CD45, CD33), up to 3 antibodies against intracellular signaling molecules, or against surface markers for an 8-color flow cytometry assay. Isotype controls or phosphopeptide blocking experiments were performed to characterize each phospho-antibody.

### Flow Cytometry Data Acquisition and Analysis

Flow cytometry data was acquired on an LSR II and/or CANTO II flow cytometer using the FACS DIVA software (BD Biosciences, San Jose, CA). All flow cytometry data were analyzed with FlowJo (TreeStar Software, Ashland, OR) or WinList (Verity House Software, Topsham, ME). Dead cells and debris were excluded by forward scatter, side scatter, and Amine Aqua Viability Dye measurement. Leukemic cells were identified as cells that fit the CD45 and CD33 versus right-angle light-scatter characteristics consistent with myeloid leukemia blasts and that lacked the characteristics of mature lymphocytes (CD45^+^, CD33^−^) (Figure S7) [65] Healthy bone marrow myeloblasts (BMMb) were identified from bone marrow mononuclear cell (BMMC) samples using right-angle light scatter and surface markers such as CD45 and CD34.

### SCNP Nomenclature and Metrics

In SCNP terminology a “signaling node” is used to refer to a proteomic readout in the presence or absence of a specific modulator. For example, the response to FLT3L treatment can be measured using p-Stat5 as a readout. That signaling node is designated “FLT3L → p-Stat5”. Several metrics (normalized assay readouts defined below and summarized in Figure 1) are applied to interpret the functionality and biology of each signaling node and are referenced following the node e.g. “FLT3L→p-Stat5 | Fold”, “G-CSF→p-Stat5 | Total” or “p-Stat5 | Basal”. These metrics were developed to measure distinct functional aspects of signaling proteins (Figure 1). To measure basal levels of signaling in the resting, unmodulated state, the “Basal” metric was applied. With modulation, the “Fold” metric identifies the inducibility or responsiveness of a protein or pathway. The “Total” metric was developed to assess the magnitude of total activated protein. It incorporates both basal and induced pathway activation and is more relevant in measuring pathways regulated by activity thresholds. For surface markers, the Relative Protein Expression (“Rel. Expression”) was used to measure the amount of surface expression and the Percent Positive (“PercentPos”) was used to quantify the frequency of cells positive for a surface marker, relative to a control antibody. For Apoptosis conditions, the percentage of cells in a two-dimensional flow plot quadrant “Quad” region e.g. the p-Chk2-,c-PARP+ quadrant defined by low levels of p-Chk2 (measuring DNA damage response) and high levels of caspase product cleaved-PARP were used to quantify levels of cellular apoptosis in response to cytotoxic drugs.

Signaling responses for the node | metric PMA→ p-Erk | Fold (a pharmacological stimulus which bypasses the need for specific surface receptors but still requires intracellular signal transduction) were ≥0.40 for 33/34 samples in study one and 82/83 samples in study two suggesting that the majority of samples were capable of induced signaling responses.

### Association between Signaling Nodes and FLT 3 Receptor Mutational Status – Univariate Analysis

All signaling nodes (Table S1) were independently tested for their ability to classify patients based on their FLT3 receptor mutational status (ITD or WT). Due to the small sample size and non-normal distribution of some node/metrics, based on visual inspection, both Student t-test and Wilcoxon p values were computed. The area under the curve of the receiver operator characteristic (AUC_ROC_) [66]–[68] was computed to assess classification accuracy of each node.

### Correlations between Node/Metrics

Pearson correlation coefficients were computed between all pairs of signaling nodes.

### Association between Multiple Node/Metric and FLT3 Mutational Status – Multivariate Analysis

All possible pairs of the 80 node/metrics that were considered to be stratifying via univariate analysis (AUC ≥0.7 or lowest p≤0.05) were evaluated for their ability to complement each other in stratifying the FLT3 receptor mutational status. A logistic regression model of the form
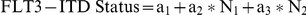
was built for each pair. a_1_ is the intercept for the model. N_1_ and N_2_ are the first and the second node/metrics respectively. The coefficients a_1_ and a_2_ are tested for their significance for being different from zero. Models in which both the nodes/metrics have a significant p-value (slope  =  0) are considered to be the nodes/metrics combinations with improved predictive value. All pairs for which the p-values were significant and the AUC for the model was greater than 0.90 (the highest AUC for an individual node/metric) were examined further.

### Outlier Analysis by Signaling

For each node /metric that stratified FLT3-ITD from FLT3-WT the list of outlier donors that are outside of 1.5 times the inter-quartile range was created. In particular, the outliers from one class (e.g. FLT-3-ITD) that are more similar to the donors in the second class (e.g. FLT-3- WT) were identified and summarized in a table together with their clinical characteristics.

## Supporting Information

Figure S1 Survival curves. Survival curves in weeks for FLT3-ITD samples (dotted line) and FLT3-WT samples (solid line) are shown for A) Study 1 and B) Study 2.
(0.59 MB TIF)

Figure S2 Assessment of false discovery rate. Expected versus actual numbers of stratifying node/metrics at specific P-value thresholds (for FLT3-WT and FLT3-ITD stratification) demonstrate significantly higher numbers of actual stratifying node/metrics observed in A) Study 1 and B) Study 2 than would be found by chance alone.
(0.77 MB TIF)

Figure S3 Basal PI3K and Jak/Stat activity in FLT3-WT and FLT3-ITD samples. A) Box and whisker plots for basal levels of p-S6, p-Erk, p-Akt in Study 1 (left panels) and Study 2 (right panels). Similar basal levels of p-S6, p-Erk, p-Akt were observed in FLT3-WT and FLT3-ITD samples. B) Box and whisker plots for basal levels of p-Stat1, p-Stat3 and p-Stat5 in Study 1 (left panels) and Study 2 (right panels). Higher levels of basal p-Stat1, p-Stat3, p-Stat5 were observed in FLT3-ITD samples in Study 1 but not Study 2. Note: Absolute values are not comparable between studies due to different experimental configurations.
(3.30 MB TIF)

Figure S4 Additional FLT3L induced signaling readouts. Box and whisker plots for FLT3L induced p-Akt, p-Erk, and p-CREB in Study 1 (left panels) and p-Akt, and p-Erk in Study 2 (right panels). FLT3-ITD samples demonstrated generally lower FLT3L induced p-Akt, p-Erk, and p-CREB compared to FLT3-WT samples. Note: Absolute values are not comparable between studies due to different experimental configurations.
(1.73 MB TIF)

Figure S5 IL-27 and FLT3L signaling in FLT3-ITD and NPM1 molecular subgroups. A) Shown are box and whisker plots of FLT3L induced p-S6, p-Erk and p-Akt (Panel A) and for IL-27 induced p-Stat responses (Panel B) for Study 1 (upper rows) and Study 2 (lower rows) in molecular subgroups. FLT3 NPM1 molecular subgroups are coded by shape: FLT3-ITD, NPM1 mutated (circles); FLT3-ITD, NPM1-WT (squares); FLT3 WT, NPM1 mutated (+ signs); FLT3 WT, NPM1 WT (X signs). For both IL-27 induced Jak/Stat signaling and FLT3L induced PI3K and Raf signaling, the most variability and largest range of response were observed within FLT3-WT NPM1-WT AML samples. In contrast, more homogenous signaling responses were observed in samples with defined molecular alterations (i.e., FLT3-ITD or NPM1 mutated).
(0.26 MB TIF)

Figure S6 Apoptosis nodes predict CR duration within FLT3-WT samples. A) Descriptive statistics for the ability of apoptosis nodes (Ara/Dauno → c-PARP| Fold, Etoposide → c-PARP| Fold) to stratify patients with CR duration of <2 years and patients with CR duration >2 years. P values, AUC_ROC_ statistics, mean value of apoptosis responses (Mean) and number (N) of patients per group are shown. B) Examples of apoptosis responses from FLT3-WT samples with CR Duration <2 years (left) and CR duration >2 years (right) are shown by overlaid histograms of cleaved PARP from unmodulated/basal (gray) and Ara/Dauno treated (black) conditions. Within FLT3-WT AML samples, apoptosis nodes Ara-C/Daunorubicin → c-PARP and Etoposide → c-PARP demonstrate higher induced apoptotic response in Complete Continuous Remission (CCR) patients with CR duration >2 years than in CR patients whose remission was <2 years.
(0.11 MB TIF)

Figure S7 Example of gating analysis to define leukemic blast population. Flow cytometry plots and sequential gating scheme. A) Flow cytometry dot plot indicating how non-cellular debris were excluded using a FSC and SSC gate. B) Flow cytometry dot plot indicating how non-viable cells were excluded with a SSC and Aqua viability dye gate. This gating scheme ensured that only from live cells were analyzed for signaling responses. C) Flow cytometry dot plot indicating how lymphocytes were excluded and myeloid leukemic blasts included using additional surface markers (i.e., CD45, CD33). Note: In the subset of AML samples with phenotypically mature blasts (CD34- CD11b++, CD33++) a small number of mature monocytes may also be included in this analysis, however, due to the abnormally high percentage of myeloid cells in these patients, the population analyzed likely contains predominately leukemic cells. Unfortunately, the side scatter properties of mature monocytes which clearly distinguish these cells from leukemic blasts are compromised by the fixation and permeabilization techniques used.
(0.92 MB TIF)

Table S1 List of nodes tested.
(0.07 MB PDF)

Table S2 Variance in signaling between healthy BMMb and FLT3 NPM1 molecular subgroups.
(0.03 MB PDF)

Table S3 Top-ranking nodes stratifying ITD from WT in Study 1 (univariate analysis).
(0.09 MB PDF)

Table S4 Top ranking nodes stratifying ITD from WT in Study 2 (univariate analysis).
(0.08 MB PDF)

Table S5 Pearson correlations between stratifying nodes for FLT3-ITD vs. FLT3-WT in Study 1.
(0.21 MB PDF)

Table S6 Combinations/pairs of nodes from linear regression analysis improve stratification of FLT3-ITD and WT samples in Study 1.
(0.08 MB PDF)

Table S7 Clinical characteristics of cytogenetically normal (CN) AML patient samples.
(0.07 MB PDF)

Table S8 Clinical characteristics of FLT3-WT vs. FLT3-ITD signaling outliers (Study 2).
(0.06 MB PDF)

Table S9 List of modulators, reagents and technical conditions.
(0.08 MB PDF)

## References

[1] Dosil M, Wang S, Lemischka IR (1993). Mitogenic signalling and substrate specificity of the Flk2/Flt3 receptor tyrosine kinase in fibroblasts and interleukin 3-dependent hematopoietic cells.. Mol Cell Biol.

[2] Rusten LS, Lyman SD, Veiby OP, Jacobsen SE (1996). The FLT3 ligand is a direct and potent stimulator of the growth of primitive and committed human CD34+ bone marrow progenitor cells in vitro.. Blood.

[3] Kayser S, Schlenk RF, Londono MC, Breitenbuecher F, Wittke K (2009). Insertion of FLT3 internal tandem duplication in the tyrosine kinase domain-1 is associated with resistance to chemotherapy and inferior outcome.. Blood.

[4] Krause DS, Van Etten RA (2005). Tyrosine kinases as targets for cancer therapy.. N Engl J Med.

[5] Whitman SP, Archer KJ, Feng L, Baldus C, Becknell B (2001). Absence of the wild-type allele predicts poor prognosis in adult de novo acute myeloid leukemia with normal cytogenetics and the internal tandem duplication of FLT3: a cancer and leukemia group B study.. Cancer Res.

[6] Kottaridis PD, Gale RE, Frew ME, Harrison G, Langabeer SE (2001). The presence of a FLT3 internal tandem duplication in patients with acute myeloid leukemia (AML) adds important prognostic information to cytogenetic risk group and response to the first cycle of chemotherapy: analysis of 854 patients from the United Kingdom Medical Research Council AML 10 and 12 trials.. Blood.

[7] Thiede C, Steudel C, Mohr B, Schaich M, Schakel U (2002). Analysis of FLT3-activating mutations in 979 patients with acute myelogenous leukemia: association with FAB subtypes and identification of subgroups with poor prognosis.. Blood.

[8] Frohling S, Schlenk RF, Breitruck J, Benner A, Kreitmeier S (2002). Prognostic significance of activating FLT3 mutations in younger adults (16 to 60 years) with acute myeloid leukemia and normal cytogenetics: a study of the AML Study Group Ulm.. Blood.

[9] Gale RE, Hills R, Kottaridis PD, Srirangan S, Wheatley K (2005). No evidence that FLT3 status should be considered as an indicator for transplantation in acute myeloid leukemia (AML): an analysis of 1135 patients, excluding acute promyelocytic leukemia, from the UK MRC AML10 and 12 trials.. Blood.

[10] Beran M, Luthra R, Kantarjian H, Estey E (2004). FLT3 mutation and response to intensive chemotherapy in young adult and elderly patients with normal karyotype.. Leuk Res.

[11] Meshinchi S, Woods WG, Stirewalt DL, Sweetser DA, Buckley JD (2001). Prevalence and prognostic significance of Flt3 internal tandem duplication in pediatric acute myeloid leukemia.. Blood.

[12] Kondo M, Horibe K, Takahashi Y, Matsumoto K, Fukuda M (1999). Prognostic value of internal tandem duplication of the FLT3 gene in childhood acute myelogenous leukemia.. Med Pediatr Oncol.

[13] Iwai T, Yokota S, Nakao M, Okamoto T, Taniwaki M (1999). Internal tandem duplication of the FLT3 gene and clinical evaluation in childhood acute myeloid leukemia. The Children's Cancer and Leukemia Study Group, Japan.. Leukemia.

[14] Kiyoi H, Naoe T, Nakano Y, Yokota S, Minami S (1999). Prognostic implication of FLT3 and N-RAS gene mutations in acute myeloid leukemia.. Blood.

[15] Moreno I, Martin G, Bolufer P, Barragan E, Rueda E (2003). Incidence and prognostic value of FLT3 internal tandem duplication and D835 mutations in acute myeloid leukemia.. Haematologica.

[16] Sheikhha MH, Awan A, Tobal K, Liu Yin JA (2003). Prognostic significance of FLT3 ITD and D835 mutations in AML patients.. Hematol J.

[17] Marcucci G, Mrozek K, Bloomfield CD (2005). Molecular heterogeneity and prognostic biomarkers in adults with acute myeloid leukemia and normal cytogenetics.. Curr Opin Hematol.

[18] Dohner H, Estey EH, Amadori S, Appelbaum FR, Buchner T (2010). Diagnosis and management of acute myeloid leukemia in adults: recommendations from an international expert panel, on behalf of the European LeukemiaNet.. Blood.

[19] Meshinchi S, Stirewalt DL, Alonzo TA, Boggon TJ, Gerbing RB (2008). Structural and numerical variation of FLT3/ITD in pediatric AML.. Blood.

[20] Stirewalt DL, Kopecky KJ, Meshinchi S, Engel JH, Pogosova-Agadjanyan EL (2006). Size of FLT3 internal tandem duplication has prognostic significance in patients with acute myeloid leukemia.. Blood.

[21] Gale RE, Green C, Allen C, Mead AJ, Burnett AK (2008). The impact of FLT3 internal tandem duplication mutant level, number, size, and interaction with NPM1 mutations in a large cohort of young adult patients with acute myeloid leukemia.. Blood.

[22] Baldus CD, Thiede C, Soucek S, Bloomfield CD, Thiel E (2006). BAALC expression and FLT3 internal tandem duplication mutations in acute myeloid leukemia patients with normal cytogenetics: prognostic implications.. J Clin Oncol.

[23] Dohner K, Schlenk RF, Habdank M, Scholl C, Rucker FG (2005). Mutant nucleophosmin (NPM1) predicts favorable prognosis in younger adults with acute myeloid leukemia and normal cytogenetics: interaction with other gene mutations.. Blood.

[24] Schnittger S, Schoch C, Kern W, Mecucci C, Tschulik C (2005). Nucleophosmin gene mutations are predictors of favorable prognosis in acute myelogenous leukemia with a normal karyotype.. Blood.

[25] Verhaak RG, Goudswaard CS, van Putten W, Bijl MA, Sanders MA (2005). Mutations in nucleophosmin (NPM1) in acute myeloid leukemia (AML): association with other gene abnormalities and previously established gene expression signatures and their favorable prognostic significance.. Blood.

[26] Green CL, Koo KK, Hills RK, Burnett AK, Linch DC (2010). Prognostic significance of CEBPA mutations in a large cohort of younger adult patients with acute myeloid leukemia: impact of double CEBPA mutations and the interaction with FLT3 and NPM1 mutations.. J Clin Oncol.

[27] Wouters BJ, Lowenberg B, Erpelinck-Verschueren CA, van Putten WL, Valk PJ (2009). Double CEBPA mutations, but not single CEBPA mutations, define a subgroup of acute myeloid leukemia with a distinctive gene expression profile that is uniquely associated with a favorable outcome.. Blood.

[28] Schlenk RF, Dohner K, Krauter J, Frohling S, Corbacioglu A (2008). Mutations and treatment outcome in cytogenetically normal acute myeloid leukemia.. N Engl J Med.

[29] Tang JL, Hou HA, Chen CY, Liu CY, Chou WC (2009). AML1/RUNX1 mutations in 470 adult patients with de novo acute myeloid leukemia: prognostic implication and interaction with other gene alterations.. Blood.

[30] Rosnet O, Buhring HJ, deLapeyriere O, Beslu N, Lavagna C (1996). Expression and signal transduction of the FLT3 tyrosine kinase receptor.. Acta Haematol.

[31] Lavagna-Sevenier C, Marchetto S, Birnbaum D, Rosnet O (1998). FLT3 signaling in hematopoietic cells involves CBL, SHC and an unknown P115 as prominent tyrosine-phosphorylated substrates.. Leukemia.

[32] Zhang S, Broxmeyer HE (2000). Flt3 ligand induces tyrosine phosphorylation of gab1 and gab2 and their association with shp-2, grb2, and PI3 kinase.. Biochem Biophys Res Commun.

[33] Zhang S, Mantel C, Broxmeyer HE (1999). Flt3 signaling involves tyrosyl-phosphorylation of SHP-2 and SHIP and their association with Grb2 and Shc in Baf3/Flt3 cells.. J Leukoc Biol.

[34] Grundler R, Miething C, Thiede C, Peschel C, Duyster J (2005). FLT3-ITD and tyrosine kinase domain mutants induce 2 distinct phenotypes in a murine bone marrow transplantation model.. Blood.

[35] Hayakawa F, Towatari M, Kiyoi H, Tanimoto M, Kitamura T (2000). Tandem-duplicated Flt3 constitutively activates STAT5 and MAP kinase and introduces autonomous cell growth in IL-3-dependent cell lines.. Oncogene.

[36] Mizuki M, Fenski R, Halfter H, Matsumura I, Schmidt R (2000). Flt3 mutations from patients with acute myeloid leukemia induce transformation of 32D cells mediated by the Ras and STAT5 pathways.. Blood.

[37] Srinivasa SP, Doshi PD (2002). Extracellular signal-regulated kinase and p38 mitogen-activated protein kinase pathways cooperate in mediating cytokine-induced proliferation of a leukemic cell line.. Leukemia.

[38] Choudhary C, Schwable J, Brandts C, Tickenbrock L, Sargin B (2005). AML-associated Flt3 kinase domain mutations show signal transduction differences compared with Flt3 ITD mutations.. Blood.

[39] Irish JM, Hovland R, Krutzik PO, Perez OD, Bruserud O (2004). Single cell profiling of potentiated phospho-protein networks in cancer cells.. Cell.

[40] Meshinchi S, Appelbaum FR (2009). Structural and functional alterations of FLT3 in acute myeloid leukemia.. Clin Cancer Res.

[41] Obermann EC, Arber C, Jotterand M, Tichelli A, Hirschmann P (2010). Expression of pSTAT5 predicts FLT3 internal tandem duplications in acute myeloid leukemia.. Ann Hematol.

[42] Irish JM, Kotecha N, Nolan GP (2006). Mapping normal and cancer cell signalling networks: towards single-cell proteomics.. Nat Rev Cancer.

[43] Krutzik PO, Nolan GP (2006). Fluorescent cell barcoding in flow cytometry allows high-throughput drug screening and signaling profiling.. Nat Methods.

[44] Sachs K, Perez O, Pe'er D, Lauffenburger DA, Nolan GP (2005). Causal protein-signaling networks derived from multiparameter single-cell data.. Science.

[45] Covey TM, Putta S, Cesano A (2010). Single Cell Network Profiling (SCNP): Mapping Drug and Target Interactions.. Assay Drug Dev Technol.

[46] Kornblau SM, Minden MD, Rosen DB, Putta S, Cohen A (2010). Dynamic single-cell network profiles in acute myelogenous leukemia are associated with patient response to standard induction therapy.. Clin Cancer Res.

[47] Lyman SD, Jacobsen SE (1998). c-kit ligand and Flt3 ligand: stem/progenitor cell factors with overlapping yet distinct activities.. Blood.

[48] Kikushige Y, Yoshimoto G, Miyamoto T, Iino T, Mori Y (2008). Human Flt3 is expressed at the hematopoietic stem cell and the granulocyte/macrophage progenitor stages to maintain cell survival.. J Immunol.

[49] Touw IP, van de Geijn GJ (2007). Granulocyte colony-stimulating factor and its receptor in normal myeloid cell development, leukemia and related blood cell disorders.. Front Biosci.

[50] Seita J, Asakawa M, Ooehara J, Takayanagi S, Morita Y (2008). Interleukin-27 directly induces differentiation in hematopoietic stem cells.. Blood.

[51] Bruserud O (1998). IL-4, IL-10 and IL-13 in acute myelogenous leukemia.. Cytokines Cell Mol Ther.

[52] Chalhoub N, Baker SJ (2009). PTEN and the PI3-kinase pathway in cancer.. Annu Rev Pathol.

[53] Brown M, Forsythe A (1974). Robust Tests for Equality of Variances.. Journal of the American Statistical Association,.

[54] Scholl C, Gilliland DG, Frohling S (2008). Deregulation of signaling pathways in acute myeloid leukemia.. Semin Oncol.

[55] Shah AJ, Smogorzewska EM, Hannum C, Crooks GM (1996). Flt3 ligand induces proliferation of quiescent human bone marrow CD34+CD38- cells and maintains progenitor cells in vitro.. Blood.

[56] Baker SJ, Rane SG, Reddy EP (2007). Hematopoietic cytokine receptor signaling.. Oncogene.

[57] Choudhary C, Brandts C, Schwable J, Tickenbrock L, Sargin B (2007). Activation mechanisms of STAT5 by oncogenic Flt3-ITD.. Blood.

[58] Pradhan A, Lambert QT, Reuther GW (2007). Transformation of hematopoietic cells and activation of JAK2-V617F by IL-27R, a component of a heterodimeric type I cytokine receptor.. Proc Natl Acad Sci U S A.

[59] Seedhouse C, Grundy M, Shang S, Ronan J, Pimblett H (2009). Impaired S-phase arrest in acute myeloid leukemia cells with a FLT3 internal tandem duplication treated with clofarabine.. Clin Cancer Res.

[60] Cheson BD, Bennett JM, Kopecky KJ, Buchner T, Willman CL (2003). Revised recommendations of the International Working Group for Diagnosis, Standardization of Response Criteria, Treatment Outcomes, and Reporting Standards for Therapeutic Trials in Acute Myeloid Leukemia.. J Clin Oncol.

[61] Yamamoto Y, Kiyoi H, Nakano Y, Suzuki R, Kodera Y (2001). Activating mutation of D835 within the activation loop of FLT3 in human hematologic malignancies.. Blood.

[62] Gorello P, Cazzaniga G, Alberti F, Dell'Oro MG, Gottardi E (2006). Quantitative assessment of minimal residual disease in acute myeloid leukemia carrying nucleophosmin (NPM1) gene mutations.. Leukemia.

[63] de Jonge-Peeters SD, Kuipers F, de Vries EG, Vellenga E (2007). ABC transporter expression in hematopoietic stem cells and the role in AML drug resistance.. Crit Rev Oncol Hematol.

[64] Svirnovski AI, Shman TV, Serhiyenka TF, Savitski VP, Smolnikova VV (2009). ABCB1 and ABCG2 proteins, their functional activity and gene expression in concert with drug sensitivity of leukemia cells.. Hematology.

[65] Stelzer GT, Goodpasture L, Stewart CC, Nicholson JKA (2000). Use of multiparameter flow cytometry and immunophenotyping for the diagnosis and classification of acute myeloid leukemia.. Immunophenotyping.

[66] Bewick V, Cheek L, Ball J (2004). Statistics review 13: receiver operating characteristic curves.. Crit Care.

[67] Hanley JA, McNeil BJ (1982). The meaning and use of the area under a receiver operating characteristic (ROC) curve.. Radiology.

[68] Hanley JA, McNeil BJ (1983). A method of comparing the areas under receiver operating characteristic curves derived from the same cases.. Radiology.

