# Supplementary figures and images for: Functional Characterization of FLT3 Receptor Signaling Deregulation in Acute Myeloid Leukemia by Single Cell Network Profiling (SCNP)
Source: PLoS One. 2010 Oct 27;5(10):e13543. doi: 10.1371/journal.pone.0013543 (PMC2965086; doi:10.1371/journal.pone.0013543)

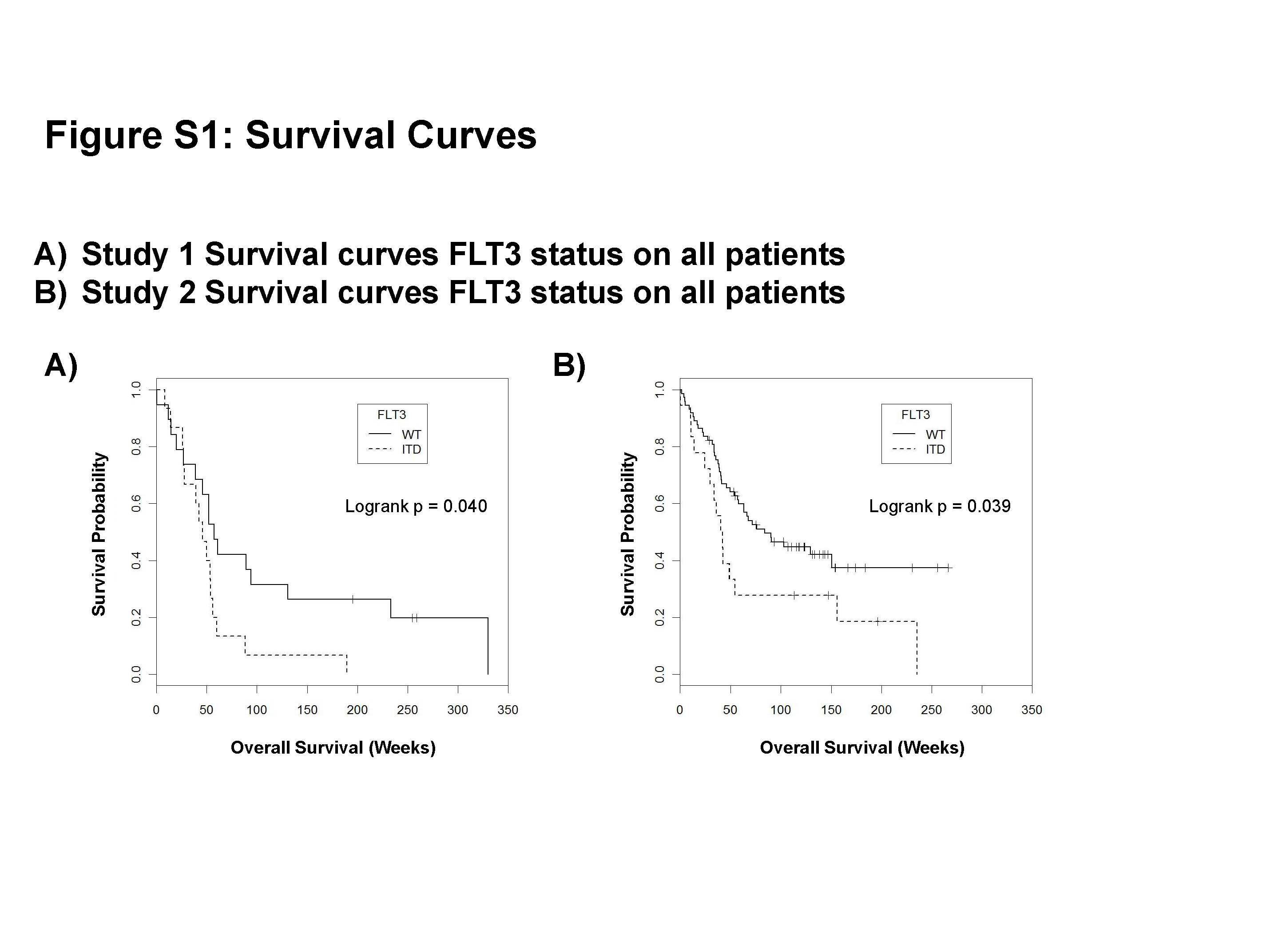

Supplement: Figure S1 — Survival curves. Survival curves in weeks for FLT3-ITD samples (dotted line) and FLT3-WT samples (solid line) are shown for A) Study 1 and B) Study 2. (0.59 MB TIF) [file pone.0013543.s001.tif]

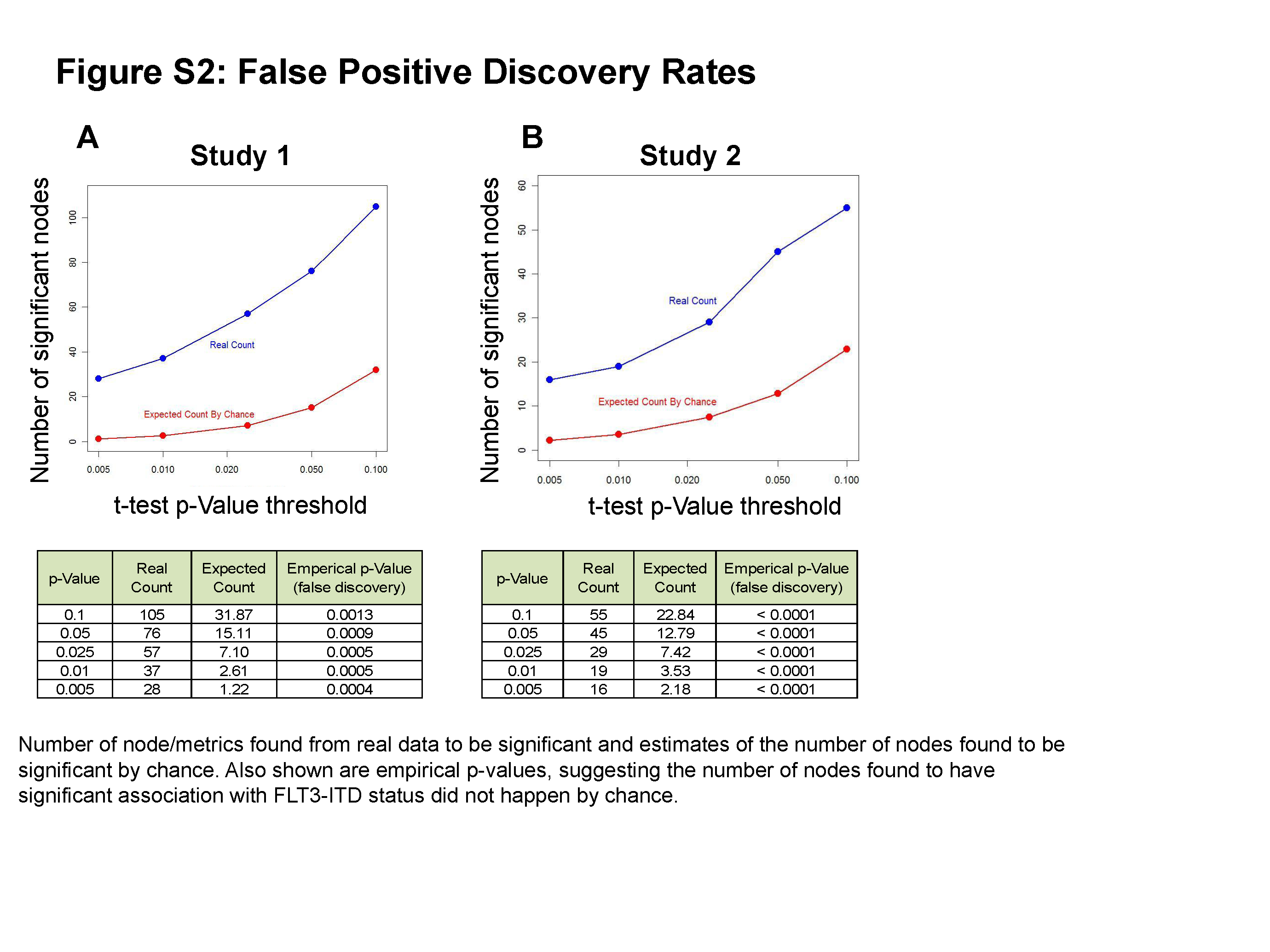

Supplement: Figure S2 — Assessment of false discovery rate. Expected versus actual numbers of stratifying node/metrics at specific P-value thresholds (for FLT3-WT and FLT3-ITD stratification) demonstrate significantly higher numbers of actual stratifying node/metrics observed in A) Study 1 and B) Study 2 than would be found by chance alone. (0.77 MB TIF) [file pone.0013543.s002.tif]

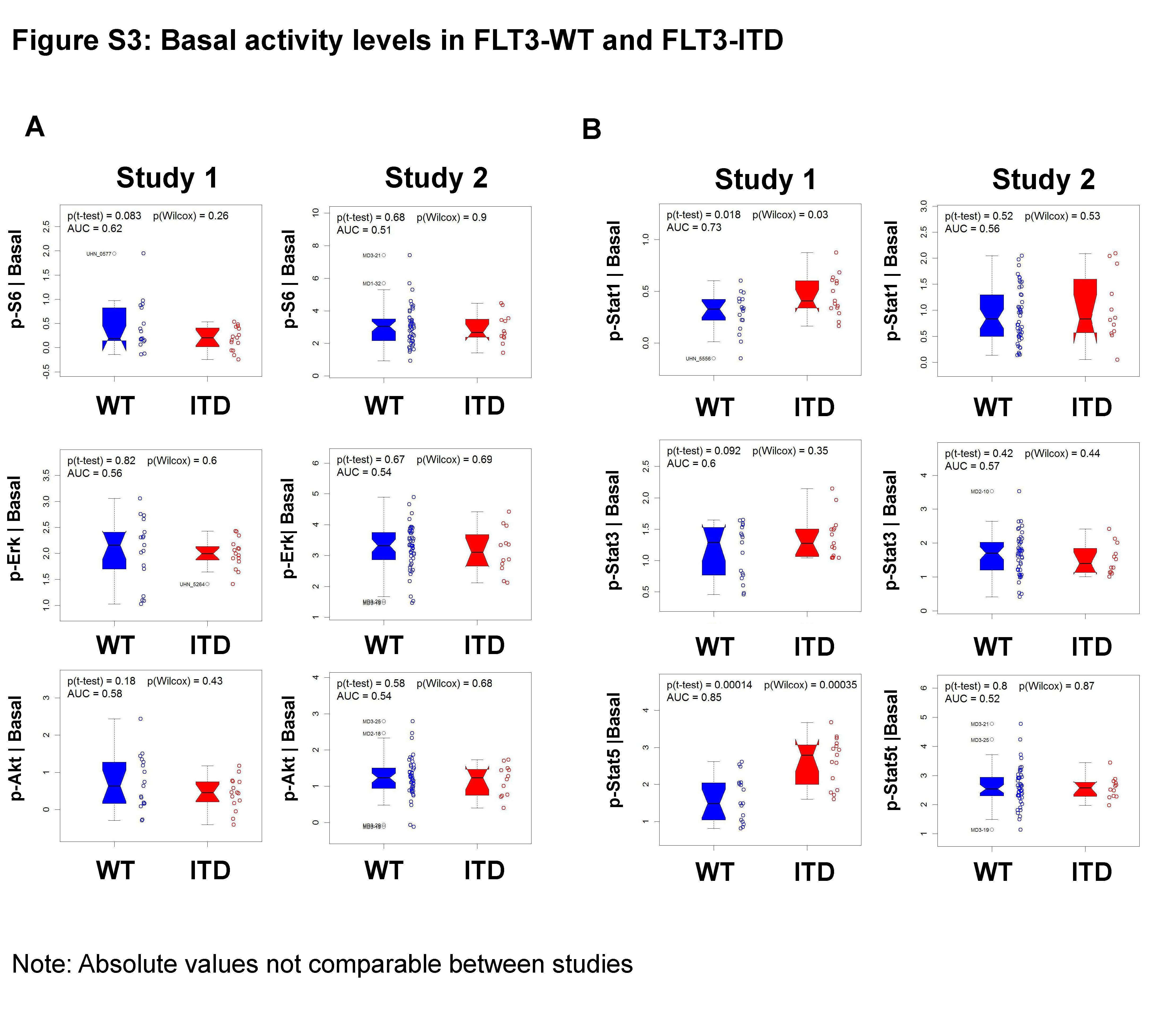

Supplement: Figure S3 — Basal PI3K and Jak/Stat activity in FLT3-WT and FLT3-ITD samples. A) Box and whisker plots for basal levels of p-S6, p-Erk, p-Akt in Study 1 (left panels) and Study 2 (right panels). Similar basal levels of p-S6, p-Erk, p-Akt were observed in FLT3-WT and FLT3-ITD samples. B) Box and whisker plots for basal levels of p-Stat1, p-Stat3 and p-Stat5 in Study 1 (left panels) and Study 2 (right panels). Higher levels of basal p-Stat1, p-Stat3, p-Stat5 were observed in FLT3-ITD samples in Study 1 but not Study 2. Note: Absolute values are not comparable between studies due to different experimental configurations. (3.30 MB TIF) [file pone.0013543.s003.tif]

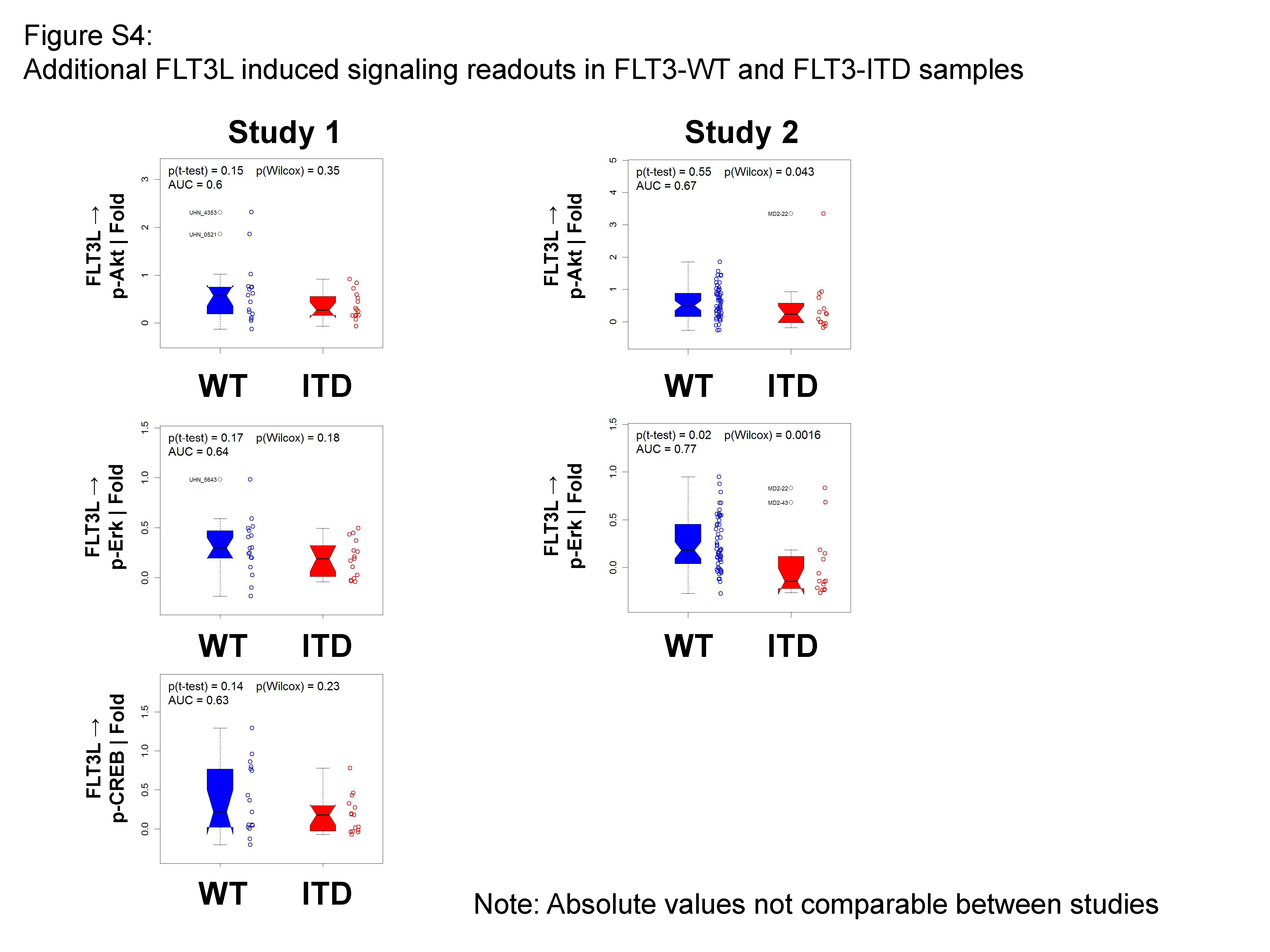

Supplement: Figure S4 — Additional FLT3L induced signaling readouts. Box and whisker plots for FLT3L induced p-Akt, p-Erk, and p-CREB in Study 1 (left panels) and p-Akt, and p-Erk in Study 2 (right panels). FLT3-ITD samples demonstrated generally lower FLT3L induced p-Akt, p-Erk, and p-CREB compared to FLT3-WT samples. Note: Absolute values are not comparable between studies due to different experimental configurations. (1.73 MB TIF) [file pone.0013543.s004.tif]

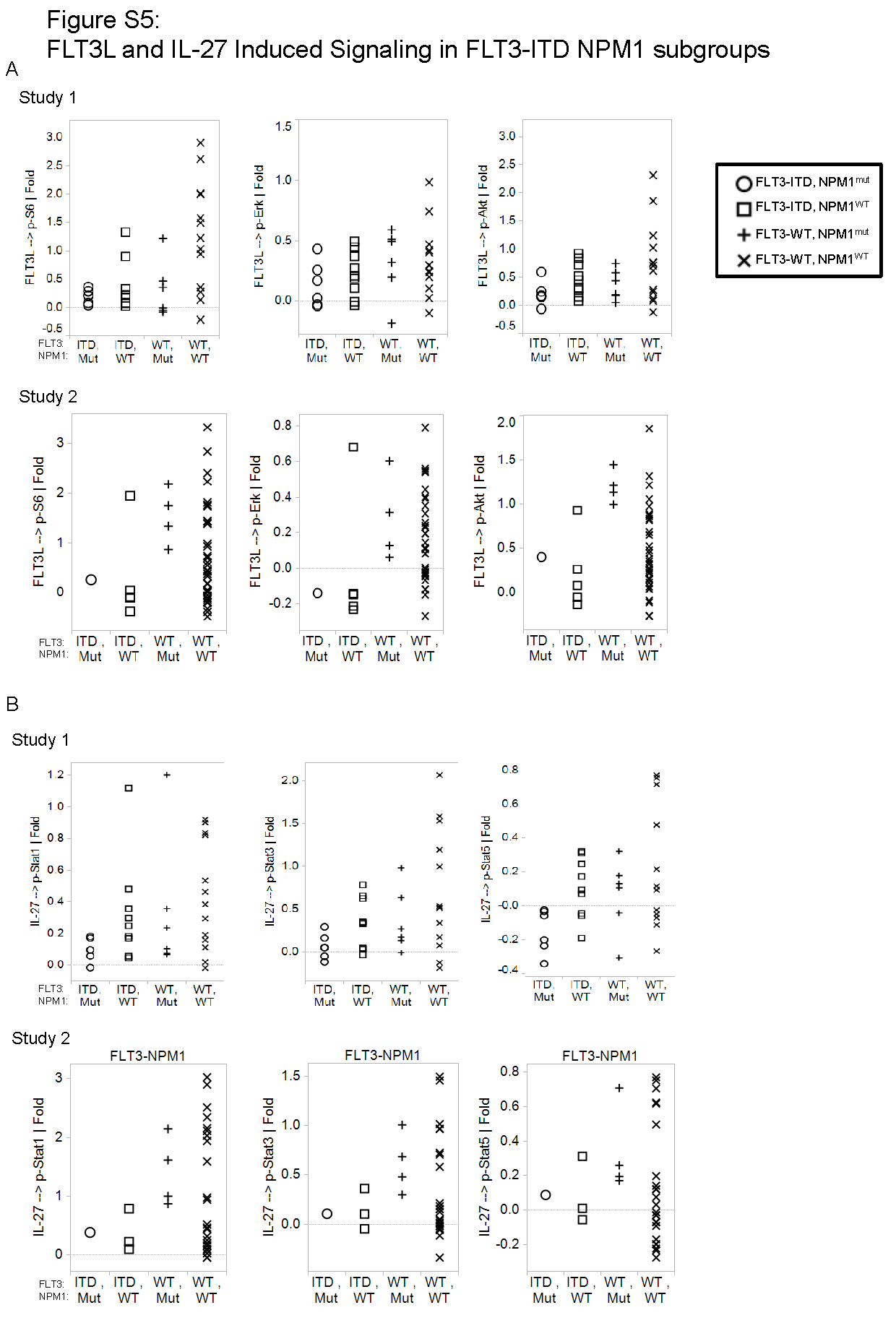

Supplement: Figure S5 — IL-27 and FLT3L signaling in FLT3-ITD and NPM1 molecular subgroups. A) Shown are box and whisker plots of FLT3L induced p-S6, p-Erk and p-Akt (Panel A) and for IL-27 induced p-Stat responses (Panel B) for Study 1 (upper rows) and Study 2 (lower rows) in molecular subgroups. FLT3 NPM1 molecular subgroups are coded by shape: FLT3-ITD, NPM1 mutated (circles); FLT3-ITD, NPM1-WT (squares); FLT3 WT, NPM1 mutated (+ signs); FLT3 WT, NPM1 WT (X signs). For both IL-27 induced Jak/Stat signaling and FLT3L induced PI3K and Raf signaling, the most variability and largest range of response were observed within FLT3-WT NPM1-WT AML samples. In contrast, more homogenous signaling responses were observed in samples with defined molecular alterations (i.e., FLT3-ITD or NPM1 mutated). (0.26 MB TIF) [file pone.0013543.s005.tif]

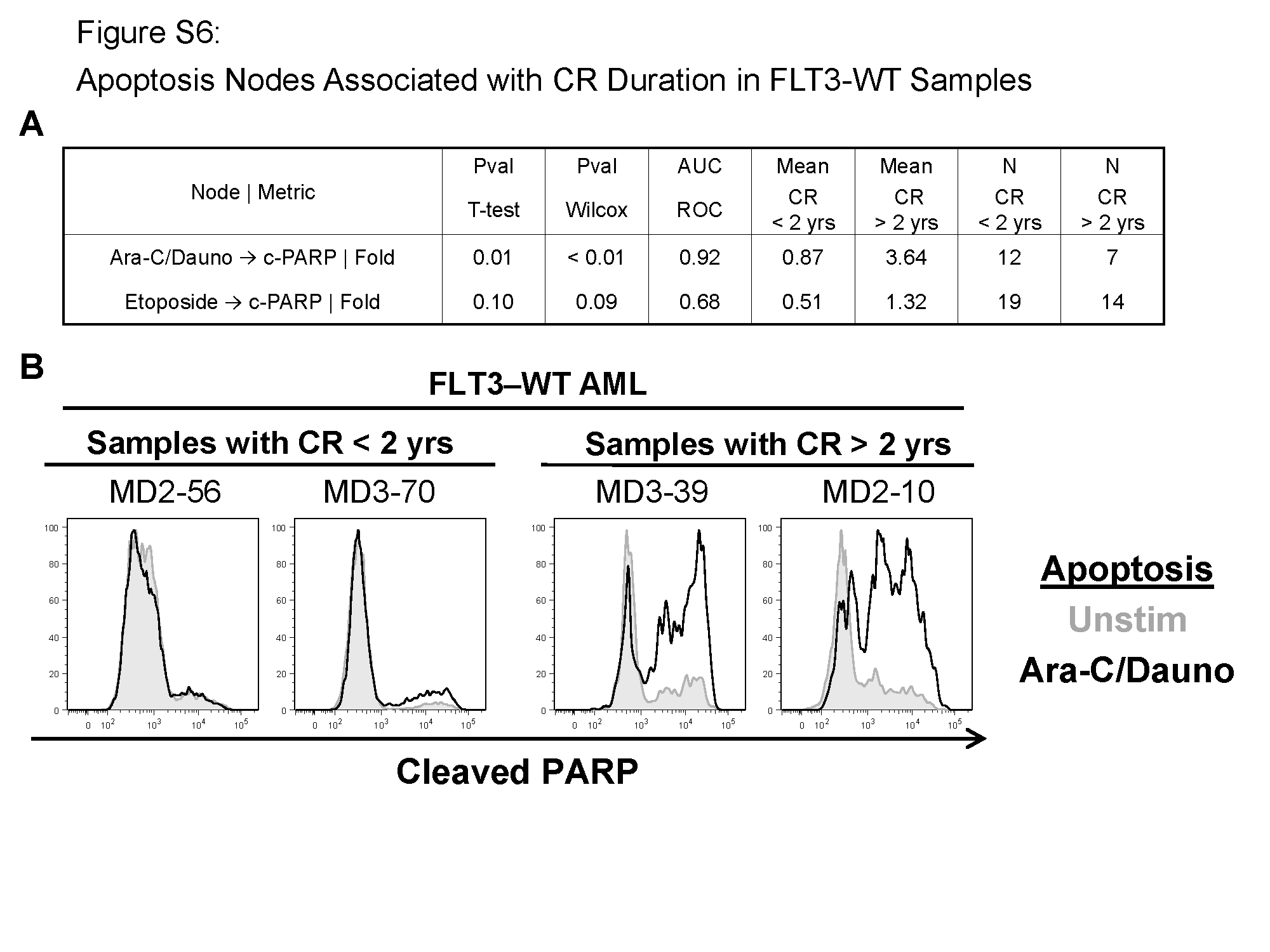

Supplement: Figure S6 — Apoptosis nodes predict CR duration within FLT3-WT samples. A) Descriptive statistics for the ability of apoptosis nodes (Ara/Dauno → c-PARP| Fold, Etoposide → c-PARP| Fold) to stratify patients with CR duration of <2 years and patients with CR duration >2 years. P values, AUCROC statistics, mean value of apoptosis responses (Mean) and number (N) of patients per group are shown. B) Examples of apoptosis responses from FLT3-WT samples with CR Duration <2 years (left) and CR duration >2 years (right) are shown by overlaid histograms of cleaved PARP from unmodulated/basal (gray) and Ara/Dauno treated (black) conditions. Within FLT3-WT AML samples, apoptosis nodes Ara-C/Daunorubicin → c-PARP and Etoposide → c-PARP demonstrate higher induced apoptotic response in Complete Continuous Remission (CCR) patients with CR duration >2 years than in CR patients whose remission was <2 years. (0.11 MB TIF) [file pone.0013543.s006.tif]

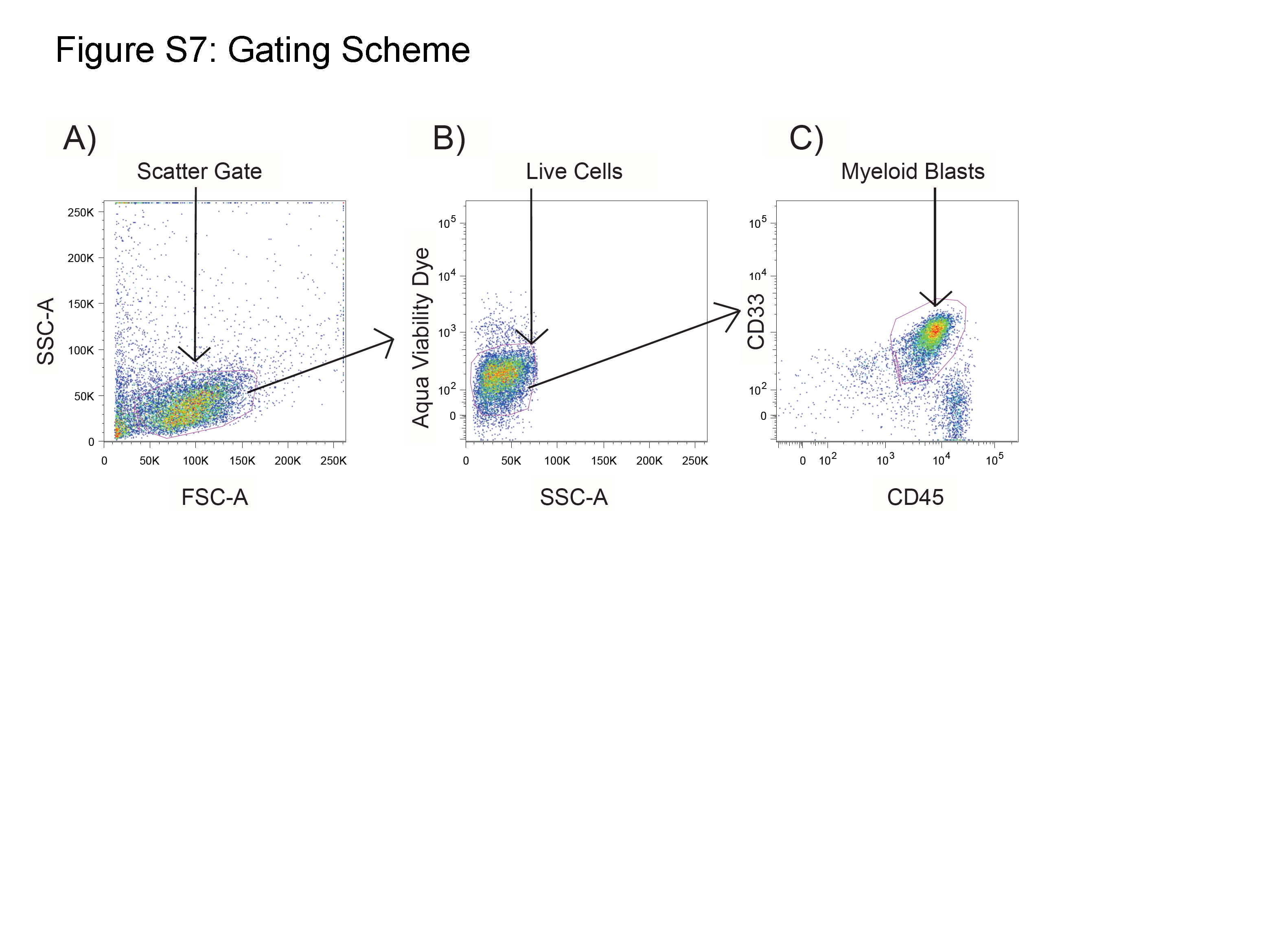

Supplement: Figure S7 — Example of gating analysis to define leukemic blast population. Flow cytometry plots and sequential gating scheme. A) Flow cytometry dot plot indicating how non-cellular debris were excluded using a FSC and SSC gate. B) Flow cytometry dot plot indicating how non-viable cells were excluded with a SSC and Aqua viability dye gate. This gating scheme ensured that only from live cells were analyzed for signaling responses. C) Flow cytometry dot plot indicating how lymphocytes were excluded and myeloid leukemic blasts included using additional surface markers (i.e., CD45, CD33). Note: In the subset of AML samples with phenotypically mature blasts (CD34- CD11b++, CD33++) a small number of mature monocytes may also be included in this analysis, however, due to the abnormally high percentage of myeloid cells in these patients, the population analyzed likely contains predominately leukemic cells. Unfortunately, the side scatter properties of mature monocytes which clearly distinguish these cells from leukemic blasts are compromised by the fixation and permeabilization techniques used. (0.92 MB TIF) [file pone.0013543.s007.tif]
