# Supplementary material for: Functional Characterization of FLT3 Receptor Signaling Deregulation in Acute Myeloid Leukemia by Single Cell Network Profiling (SCNP)
Source: PLoS One. 2010 Oct 27;5(10):e13543. doi: 10.1371/journal.pone.0013543 (PMC2965086; doi:10.1371/journal.pone.0013543)
Supplement: Table S1 — List of nodes tested. (0.07 MB PDF) [file pone.0013543.s008.pdf]

Table S1. List of nodes tested.

Biological Category	Node: Modulator	Node: Readout (antibody specificity)	Included in Study 1	Included in Study 2
CCG	CD40L	p-S6 (S235)	x	
		p-CREB (S133)	x	
		p-Erk 1/2 (T202/204)	x	
		p-p38 (T180/Y182)	x	
		p-NFkB p 65 (S529)	x	
CCG	EPO	p-Stat1 (Y701)	x	
		p-Stat3 (Y705)	x	
		p-Stat5 (Y694)	x	
CCG	FLT3L	p-S6 (S235)	x	x
		p-Erk 1/2 (T202/204)	x	x
		p-Akt (S473)	x	x
		p-CREB (S133)	x	x
		p-PLCγ2 (Y759)	x	x
		p-Stat5 (Y694)	x	x
CCG	G-CSF	p-Stat1 (Y701)	x	x
		p-Stat3 (Y705)	x	x
		p-Stat5 (Y694)	x	x
		p-S6 (S235)	x	
		p-Erk 1/2 (T202/204)	x	
		p-Akt (S473)	x	
CCG	GM-CSF	p-Stat1 (Y701)	x	x
		p-Stat3 (Y705)	x	x
		p-Stat5 (Y694)	x	x
		p-S6 (S235)	x	
		p-Erk 1/2 (T202/204)	x	
		p-Akt (S473)	x	
CCG	IFNα	p-Stat1 (Y701)	x	x
		p-Stat3 (Y705)	x	x
		p-Stat5 (Y694)	x	x
CCG	IFNγ	p-Stat1 (Y701)	x	x
		p-Stat3 (Y705)	x	x
		p-Stat5 (Y694)	x	x
CCG	IGF-1	p-S6 (S235)	x	
		p-CREB (S133)	x	
		p-Erk 1/2 (T202/204)	x	
		p-PLCγ2 (Y759)	x	
		p-Stat5 (Y694)	x	
CCG	IL-10	p-Stat1 (Y701)	x	x
		p-Stat3 (Y705)	x	x
		p-Stat5 (Y694)	x	x
CCG	IL-27	p-Stat1 (Y701)	x	x
		p-Stat3 (Y705)	x	x
		p-Stat5 (Y694)	x	x
		p-S6 (S235)	x	
		p-CREB (S133)	x	
		p-Erk 1/2 (T202/204)	x	

Table S1. List of nodes tested.

Biological Category	Node: Modulator	Node: Readout (antibody specificity)	Included in Study 1	Included in Study 2
CCG	IL-3	p-Stat1 (Y701)	x	x
		p-Stat3 (Y705)	x	x
		p-Stat5 (Y694)	x	x
		p-S6 (S235)	x	
		p-CREB (S133)	x	
		p-Erk 1/2 (T202/204)	x	
CCG	IL-4	p-Stat6 (Y641)	x	x
		p-Stat5 (Y694)	x	x
CCG	IL-6	p-Stat1 (Y701)	x	x
		p-Stat3 (Y705)	x	x
		p-Stat5 (Y694)	x	x
		p-S6 (S235)	x	
		p-CREB (S133)	x	
		p-Erk 1/2 (T202/204)	x	
CCG	LPS	p-p38 (T180/Y182)	x	x
		p-Erk 1/2 (T202/204)	x	x
		p-NFkB p 65 (S529)	x	x
CCG	M-CSF	p-S6 (S235)	x	x
		p-Erk 1/2 (T202/204)	x	x
		p-Akt (S473)	x	x
		p-CREB (S133)	x	
		p-PLCγ2 (Y759)	x	
		p-Stat5 (Y694)	x	
CCG	PMA	p-S6 (S235)	x	x
		p-CREB (S133	x	x
		p-Erk 1/2 (T202/204)	x	x
CCG	SCF	p-S6 (S235)	x	x
		p-Erk 1/2 (T202/204)	x	x
		p-Akt (S473)	x	x
		p-CREB (S133)	x	x
		p-PLCγ2 (Y759)	x	x
		p-Stat5 (Y694)	x	x
CCG	SDF-1α	p-CREB (S133)		x
		p-S6 (S235)	x	
		p-Erk 1/2 (T202/204)	x	
		p-Akt (S473)	x	x
CCG	Thapsigargin	p-S6 (S235)	x	x
		p-CREB (S133	x	x
		p-Erk 1/2 (T202/204)	x	x
CCG	TNFα	p-p38 (T180/Y182)	x	x
		p-Erk 1/2 (T202/204)	x	x
		p-NFkB p 65 (S529)	x	x
Phosphatase & ROS	H_2O_2	p-Akt (S473)	x	x
		p-Stat1 (Y701)	x	
		p-Stat3 (Y705)	x	
		p-Stat5 (Y694)	x	
		p-Lck (Y505)	x	
		p-PLCγ2 (Y759)	x	x
		p-SLP76 (Y128)	x	x
		p-S6 (S235)	x	
		p-Erk 1/2 (T202/204)	x	

Table S1. List of nodes tested.

Biological Category	Node: Modulator	Node: Readout (antibody specificity)	Included in Study 1	Included in Study 2
Phosphatase & ROS	H_2O_2 + IFNα	p-Stat1 (Y701)	x	
		p-Stat3 (Y705)	x	
		p-Stat5 (Y694)	x	
Phosphatase & ROS	H_2O_2 + SCF	p-Lck (Y505)	x	
		p-PLCγ2 (Y759)	x	
		p-SLP76 (Y128)	x	
		p-S6 (S235)	x	
		p-Erk 1/2 (T202/204)	x	
		p-Akt (S473)	x	
Surface Markers	None/ Phenotypic Stain	CXCR4	x	x
		MRP1		x
		ABCG2	x	x
		FLT3 Receptor	x	x
		cKit	x	x
		EPO-R	x	
		MCSF-R	x	
		TNF-R	x	
		CD40	x	
Apoptosis	Ara-C + Dauno	c-PARP		x
		p-Chk2 (T68)		x
		Dauno		x
Apoptosis	Etoposide	c-PARP	x	x
		p-Chk2 (T68)	x	x
		BCL-2	x	
		c-Caspase 3	x	
Apoptosis	Etoposide + ZVAD	BCL-2	x	
		c-PARP	x	
		p-Chk2 (T68)	x	
		c-Caspase 3	x	
Apoptosis	Stauroporine	Cytochrome C		x
		BCL-2	x	
		c-PARP	x	x
		c-Caspase 8	x	x
		c-Caspase 3	x	
Apoptosis		Cytochrome C		x
	Staurosporine + ZVAD	BCL-2	x	
		c-PARP	x	x
		c-Caspase 8	x	x
		c-Caspase 3	x	

Each modulator and read-out combination is a node. Unmodulated, basal levels were also measured. In Study 1, there were 18 basal, 121 modulated, and 8 surface markers for a total node count of 147. In Study 2, there were 16 basal, 69 modulated, and 5 surface markers for a total node count of 90.

Akt indicates protein kinase B; APC, allophyco-cyanin; Ara-C, cytarabine; ABCG2, ATP-binding cassette, sub-family G, member 2; BCL, B-cell Leukemia/Lymphoma; CD, cluster of differentiation; c-, cleaved-; CCG, cytokine, chemokine, growth factor; C-kit, CD117; CREB, cAMP response element binding; CXCR, CXC chemokine receptor; EPO, erythropoietin; Erk, Extracellular signal-regulated kinase; FITC, fluorescein isothiocyanate; FLT3, fms-like tyrosine kinase; G-CSF, granulocyte colony stimulating factor; GM-CSF, granulocyte macrophage colony stimulating factor; H_2O_2, hydrogen peroxide; IFN, interferon; IGF, insulin-like growth factor; IL, interleukin; M-CSF, macrophage colony stimulating factor; MDR, p-glycoprotein; NFkB, Nuclear Factor-Kappa B; p-, phospho-; p38, map kinase family protein 38; PARP, poly (ADP-ribose) polymerase; PE, phycoerythrin; PLCγ2, phospholipase c-gamma; S6, ribosomal protein S6; SCF, stem cell factor; SDF, stromal cell derived factor; Stat, signal transducer and activator of transcription; Stauro, staurosporine; TNF, tumor necrosis factor; ZVAD, ZVAD-FMK caspase inhibitor
