# Supplementary material for: Functional Characterization of FLT3 Receptor Signaling Deregulation in Acute Myeloid Leukemia by Single Cell Network Profiling (SCNP)
Source: PLoS One. 2010 Oct 27;5(10):e13543. doi: 10.1371/journal.pone.0013543 (PMC2965086; doi:10.1371/journal.pone.0013543)
Supplement: Table S2 — Variance in signaling between healthy BMMb and FLT3 NPM1 molecular subgroups. (0.03 MB PDF) [file pone.0013543.s009.pdf]

Table S2A. Variance in signaling between FLT3-WT samples, FLT3-ITD samples, and Healthy BMMb.

Node | Metric:	Standard Deviation			Levene's test p-value		
	Healthy BMMb	FLT3-ITD AML	FLT3-WT AML	FLT3-WT vs Healthy BMMb	FLT3-WT vs FLT3-ITD	FLT3-ITD vs Healthy BMMb
FLT3L→p-S6 | Fold	0.179	0.343	0.921	0.0030	0.0004	0.6499

Levene's test assesses the equality of variances in different samples
A significant p value in a Levene's test infers unequal variance between groups
Variance of signaling was compared between AML samples from Study 1 and Healthy BMMb from a separate study.

Table S2B. Variance in signaling between FLT3 NPM1 molecular subgroups.

Node | Metric: FLT3L→p-S6 | Fold

Molecular Subgroup:		Levene's test p-value			
		FLT3-ITD NPM1-Mut	FLT3-ITD NPM1-WT	FLT3-WT NPM1-Mut	FLT3-WT NPM1-WT
FLT3-ITD	NPM1-Mut	1	0.267	0.08	0.007
FLT3-ITD	NPM1-WT		1	0.64	0.024
FLT3-WT	NPM1-Mut			1	0.1
FLT3-WT	NPM1-WT				1

Node | Metric:	Standard Deviation			
	FLT3-ITD NPM1-Mut	FLT3-ITD NPM1-WT	FLT3-WT NPM1-Mut	FLT3-WT NPM1-WT
FLT3L→p-S6 | Fold	0.111	0.419	0.452	0.930

Node | Metric: IL-27→p-Stat3 | Fold

Molecular Subgroup:		Levene's test p-value			
		FLT3-ITD NPM1-Mut	FLT3-ITD NPM1-WT	FLT3-WT NPM1-Mut	FLT3-WT NPM1-WT
FLT3-ITD	NPM1-Mut	1	0.137	0.216	0.036
FLT3-ITD	NPM1-WT		1	0.753	0.064
FLT3-WT	NPM1-Mut			1	0.189
FLT3-WT	NPM1-WT				1

Node | Metric:	Standard Deviation			
	FLT3-ITD NPM1-Mut	FLT3-ITD NPM1-WT	FLT3-WT NPM1-Mut	FLT3-WT NPM1-WT
IL-27→p-Stat3 | Fold	0.135	0.279	0.340	0.683

Levene's test assesses the equality of variances in different samples
A significant p value in a Levene's test infers unequal variance between groups
