# Supplementary material for: Functional Characterization of FLT3 Receptor Signaling Deregulation in Acute Myeloid Leukemia by Single Cell Network Profiling (SCNP)
Source: PLoS One. 2010 Oct 27;5(10):e13543. doi: 10.1371/journal.pone.0013543 (PMC2965086; doi:10.1371/journal.pone.0013543)
Supplement: Table S3 — Top-ranking nodes stratifying ITD from WT in Study 1 (univariate analysis). (0.09 MB PDF) [file pone.0013543.s010.pdf]

Table S3. Top ranking nodes stratifying ITD from WT in Study 1 (univariate analysis).

Biological Category	Node | Metric	AUC_{ROC}	Num. WTs/ITDs	t-test *P*	Wilcoxon *P*	Mean Value of WTs/ITDs
Apoptosis	Etoposide & ZVAD→c-Caspase-3 | Total	0.73	12 / 14	0.027	0.053	0.58 / 1.19
Apoptosis	Etoposide & ZVAD→c-PARP | Fold	0.82	14 / 14	0.009	0.003	0.89 / 2.16
Apoptosis	Etoposide & ZVAD→c-PARP | Total	0.80	14 / 14	0.001	0.006	1.40 / 2.86
Apoptosis	Etoposide & ZVAD→p-Chk2-, c-PARP + | Quad	0.73	14 / 14	0.094	0.035	0.10 / 0.16
Apoptosis	Etoposide & ZVAD→p-Chk2+, c-PARP - | Quad	0.78	14 / 14	0.012	0.012	0.46 / 0.25
Apoptosis	Etoposide & ZVAD→p-Chk2+, c-PARP + | Quad	0.77	14 / 14	0.009	0.015	0.30 / 0.49
Apoptosis	Etoposide→BCL2 | Fold	0.75	13 / 14	0.023	0.025	-0.01 / 0.12
Apoptosis	Etoposide→c-Caspase-3 | Fold	0.80	12 / 14	0.010	0.008	0.53 / 1.50
Apoptosis	Etoposide→c-Caspase-3 | Total	0.82	12 / 14	0.003	0.004	0.94 / 2.25
Apoptosis	Etoposide→c-PARP | Fold	0.83	14 / 14	0.001	0.002	0.55 / 2.08
Apoptosis	Etoposide→c-PARP | Total	0.82	14 / 14	0.001	0.003	1.06 / 2.79
Apoptosis	Etoposide→p-Chk2 | Fold	0.71	13 / 14	0.039	0.068	0.27 / -0.31
Apoptosis	Etoposide→p-Chk2-, c-PARP + | Quad	0.81	14 / 14	0.005	0.005	0.21 / 0.44
Apoptosis	Etoposide→p-Chk2+, c-PARP - | Quad	0.71	14 / 14	0.031	0.063	0.51 / 0.30
Apoptosis	Staurosporine & ZVAD→c-Caspase-3 | Fold	0.86	15 / 14	0.001	0.001	0.20 / 0.98
Apoptosis	Staurosporine & ZVAD→c-Caspase-3 | Total	0.89	15 / 14	0.001	0.000	0.46 / 1.18
Apoptosis	Staurosporine & ZVAD→c-Caspase-8 | Fold	0.90	15 / 14	0.000	0.000	-0.25 / 1.12
Apoptosis	Staurosporine & ZVAD→c-Caspase-8 | Total	0.77	15 / 14	0.009	0.014	4.92 / 5.96
Apoptosis	Staurosporine & ZVAD→c-PARP | Fold	0.93	15 / 14	0.000	0.000	0.72 / 4.05
Apoptosis	Staurosporine & ZVAD→c-PARP | Total	0.94	15 / 14	0.000	0.000	0.85 / 4.20
Apoptosis	Staurosporine→c-Caspase-3 | Fold	0.84	15 / 14	0.001	0.001	1.26 / 2.55
Apoptosis	Staurosporine→c-Caspase-3 | Total	0.82	15 / 14	0.002	0.003	1.56 / 2.72
Apoptosis	Staurosporine→c-Caspase-8 | Fold	0.85	15 / 14	0.000	0.002	1.29 / 3.24
Apoptosis	Staurosporine→c-Caspase-8 | Total	0.87	15 / 14	0.000	0.000	6.45 / 8.09
Apoptosis	Staurosporine→c-PARP | Fold	0.86	15 / 14	0.000	0.001	2.43 / 5.30
Apoptosis	Staurosporine→c-PARP | Total	0.89	15 / 14	0.000	0.000	2.56 / 5.44
CCG	CD40L→p-p38 | Total	0.68	17 / 15	0.032	0.089	0.48 / 0.19
CCG	CD40L→p-S6 | Total	0.71	17 / 15	0.048	0.040	0.54 / 0.20
CCG	EPO→p-Stat1 | Total	0.71	17 / 15	0.054	0.044	0.29 / 0.45
CCG	EPO→p-Stat5 | Total	0.87	17 / 15	0.000	0.000	1.12 / 2.16
CCG	FLT3L→p-CREB | Total	0.77	17 / 15	0.012	0.009	2.20 / 1.65
CCG	FLT3L→p-S6 | Fold	0.72	17 / 15	0.014	0.038	0.96 / 0.30
CCG	FLT3L→p-S6 | Total	0.80	17 / 15	0.003	0.003	1.46 / 0.45
CCG	FLT3L→p-Stat5 | Total	0.81	17 / 15	0.024	0.002	1.44 / 2.14
CCG	G-CSF→p-Erk | Fold	0.72	17 / 15	0.053	0.033	0.04 / 0.22
CCG	G-CSF→p-S6 | Total	0.64	17 / 15	0.034	0.176	0.54 / 0.23
CCG	G-CSF→p-Stat5 | Total	0.75	17 / 15	0.020	0.016	2.18 / 3.22
CCG	GM-CSF→p-Stat5 | Total	0.78	17 / 15	0.021	0.005	2.26 / 3.42
CCG	IFNα→p-Stat1 | Total	0.77	17 / 15	0.010	0.008	0.86 / 1.27
CCG	IFNα→p-Stat3 | Total	0.73	17 / 15	0.042	0.027	1.92 / 2.49
CCG	IFNα→p-Stat5 | Total	0.80	17 / 15	0.007	0.003	2.73 / 3.91
CCG	IFNγ→p-Stat5 | Fold	0.75	17 / 15	0.109	0.018	0.72 / 0.32
CCG	IFNγ→p-Stat5 | Total	0.77	17 / 15	0.004	0.008	1.84 / 2.68
CCG	IGF-1→p-Stat5 | Total	0.85	17 / 15	0.000	0.000	1.18 / 2.16
CCG	IL-10→p-Stat5 | Total	0.87	17 / 15	0.000	0.000	1.25 / 2.26
CCG	IL-27→p-Erk | Total	0.71	17 / 15	0.024	0.049	1.38 / 0.81
CCG	IL-27→p-S6 | Total	0.72	17 / 15	0.047	0.033	0.47 / 0.15
CCG	IL-27→p-Stat3 | Fold	0.69	17 / 15	0.029	0.076	0.64 / 0.23
CCG	IL-27→p-Stat5 | Fold	0.70	17 / 15	0.038	0.059	0.21 / 0.00
CCG	IL-27→p-Stat5 | Total	0.85	17 / 15	0.000	0.000	1.48 / 2.35
CCG	IL-3→p-S6 | Total	0.71	17 / 15	0.066	0.044	0.56 / 0.25
CCG	IL-3→p-Stat5 | Total	0.72	17 / 15	0.084	0.033	2.68 / 3.53
CCG	IL-4→p-Stat5 | Total	0.81	17 / 15	0.006	0.002	2.14 / 3.32
CCG	IL-6→p-CREB | Fold	0.73	17 / 15	0.017	0.030	-0.30 / 0.07
CCG	IL-6→p-S6 | Fold	0.73	17 / 15	0.659	0.026	0.07 / 0.09
CCG	IL-6→p-Stat5 | Total	0.84	17 / 15	0.000	0.001	1.19 / 2.18
CCG	M-CSF→p-S6 | Fold	0.76	17 / 15	0.020	0.011	-0.14 / -0.01
CCG	M-CSF→p-Stat5 | Total	0.85	17 / 15	0.000	0.000	1.30 / 2.21
CCG	p-SLP-76 | Basal	0.76	13 / 14	0.022	0.019	1.16 / -0.19
CCG	p-Stat1 | Basal	0.73	17 / 15	0.018	0.030	0.29 / 0.46
CCG	p-Stat5 | Basal	0.85	17 / 15	0.000	0.000	1.63 / 2.62
CCG	PMA→p-CREB | Fold	0.75	17 / 15	0.011	0.018	1.18 / 1.95
CCG	SCF→p-CREB | Total	0.73	17 / 15	0.040	0.027	1.96 / 1.54
CCG	SCF→p-S6 | Total	0.65	17 / 15	0.043	0.153	0.95 / 0.43
CCG	SCF→p-Stat5 | Total	0.87	17 / 15	0.000	0.000	1.26 / 2.19
CCG	Thapsigargin→p-CREB | Fold	0.75	17 / 15	0.011	0.016	0.06 / 0.74
CCG	Thapsigargin→p-CREB | Total	0.66	17 / 15	0.047	0.132	2.31 / 2.95
CCG	TNFα→p-NFkB-p65 | Fold	0.73	17 / 15	0.010	0.024	1.41 / 2.22
Phosphatase & ROS	H_2O_2 & IFNα→p-Stat1 | Total	0.80	14 / 14	0.004	0.007	1.65 / 2.19
Phosphatase & ROS	H_2O_2 & IFNα→p-Stat5 | Total	0.81	14 / 14	0.010	0.005	2.22 / 3.30
Phosphatase & ROS	H_2O_2 & SCF→p-Erk | Fold	0.91	14 / 14	0.006	0.000	0.49 / 0.08
Phosphatase & ROS	H_2O_2 & SCF→p-Erk | Total	0.72	14 / 14	0.042	0.044	2.76 / 2.28
Phosphatase & ROS	H_2O_2→p-Erk | Fold	0.73	14 / 14	0.029	0.043	0.45 / 0.03
Phosphatase & ROS	H_2O_2→p-Stat5 | Fold	0.84	14 / 14	0.008	0.002	0.18 / -0.36
Surface Markers	ABCG2 | PercentPos	0.73	16 / 14	0.052	0.031	7.14 / 8.58
Surface Markers	ABCG2 | Rel. Expression	0.74	16 / 14	0.015	0.028	0.21 / 0.38
Surface Markers	cKit | PercentPos	0.71	16 / 14	0.032	0.047	48.39 / 65.21

Node/Metrics with a t-test *P* value or Wilcoxon *P* value of ≤ 0.05 and an AUC_{ROC} of ≥ 0.6 are shown

Metrics are defined in the Materials & Methods and shown in Figure 2
