# Supplementary material for: Functional Characterization of FLT3 Receptor Signaling Deregulation in Acute Myeloid Leukemia by Single Cell Network Profiling (SCNP)
Source: PLoS One. 2010 Oct 27;5(10):e13543. doi: 10.1371/journal.pone.0013543 (PMC2965086; doi:10.1371/journal.pone.0013543)
Supplement: Table S4 — Top ranking nodes stratifying ITD from WT in Study 2 (univariate analysis). (0.08 MB PDF) [file pone.0013543.s011.pdf]

Table S4. Top ranking nodes stratifying ITD from WT in Study 2 (univariate analysis).

Biological Category	Node/Metric	AUC_{ROC}	Num. WTs/ITDs	t-test *P*	Wilcoxon *P*	Mean Value of WTs/ITDs
Apoptosis	Ara-C & Dauno→c-PARP | Total	0.76	35 / 9	0.001	0.018	3.34 / 5.13
Apoptosis	c-PARP | Basal	0.72	47 / 12	0.046	0.020	2.10 / 3.58
Apoptosis	Etoposide→c-PARP | Total	0.73	47 / 12	0.005	0.013	2.81 / 4.45
Apoptosis	p-Chk2 | Basal	0.71	47 / 12	0.062	0.024	4.53 / 4.07
Apoptosis	Staurosporine & ZVAD→c-PARP | Fold	0.82	13 / 3	0.002	0.111	1.64 / 4.35
Apoptosis	Staurosporine & ZVAD→c-PARP | Total	0.97	13 / 3	0.000	0.007	3.16 / 6.22
CCG	FLT3L→p-Akt | Fold	0.67	58 / 15	0.553	0.043	0.59 / 0.45
CCG	FLT3L→p-Erk | Fold	0.77	58 / 15	0.020	0.002	0.25 / 0.01
CCG	FLT3L→p-S6 | Fold	0.68	58 / 15	0.028	0.036	1.03 / 0.43
CCG	FLT3L→p-S6 | Total	0.65	48 / 12	0.035	0.119	4.09 / 3.27
CCG	G-CSF→p-Stat1 | Fold	0.68	49 / 12	0.002	0.057	0.30 / 0.03
CCG	G-CSF→p-Stat3 | Fold	0.69	49 / 12	0.003	0.050	1.01 / 0.29
CCG	G-CSF→p-Stat3 | Total	0.68	48 / 12	0.003	0.053	2.69 / 1.82
CCG	G-CSF→p-Stat5 | Fold	0.71	49 / 12	0.008	0.024	1.05 / 0.25
CCG	G-CSF→p-Stat5 | Total	0.70	47 / 12	0.016	0.038	3.76 / 2.86
CCG	GM-CSF→p-Stat5 | Fold	0.70	11 / 3	0.020	0.368	1.96 / 0.61
CCG	IFNα→p-Stat1 | Fold	0.78	35 / 9	0.003	0.008	2.05 / 0.97
CCG	IFNα→p-Stat1 | Total	0.73	35 / 9	0.029	0.035	2.97 / 2.09
CCG	IFNα→p-Stat3 | Fold	0.75	35 / 9	0.016	0.025	0.98 / 0.47
CCG	IFNα→p-Stat3 | Total	0.73	35 / 9	0.009	0.035	2.77 / 2.07
CCG	IFNα→p-Stat5 | Fold	0.75	35 / 9	0.031	0.020	1.68 / 0.78
CCG	IFNα→p-Stat5 | Total	0.74	35 / 9	0.047	0.030	4.44 / 3.45
CCG	IFNγ→p-Stat1 | Fold	0.79	15 / 5	0.010	0.066	1.79 / 0.88
CCG	IFNγ→p-Stat5 | Fold	0.76	15 / 5	0.022	0.098	0.71 / 0.32
CCG	IL-27→p-Stat1 | Fold	0.71	44 / 11	0.000	0.028	1.01 / 0.28
CCG	IL-27→p-Stat1 | Total	0.68	43 / 11	0.027	0.070	1.97 / 1.40
CCG	IL-27→p-Stat3 | Fold	0.73	44 / 11	0.002	0.022	0.37 / 0.04
CCG	IL-27→p-Stat3 | Total	0.67	43 / 11	0.027	0.082	2.08 / 1.61
CCG	IL-27→p-Stat5 | Fold	0.63	44 / 11	0.024	0.207	0.15 / -0.02
CCG	IL-4→p-Stat5 | Fold	1.00	7 / 2	0.037	0.056	0.36 / -0.09
CCG	M-CSF→p-Erk | Total	1.00	7 / 2	0.003	0.056	3.08 / 4.42
CCG	p-Plcγ2 | Basal	0.93	7 / 2	0.043	0.111	1.42 / 2.17
CCG	p-SLP-76 | Basal	0.68	47 / 12	0.042	0.061	1.73 / 1.27
CCG	PMA→p-CREB | Fold	0.74	35 / 9	0.016	0.027	0.76 / 0.20
CCG	PMA→p-CREB | Total	0.72	35 / 9	0.027	0.043	3.96 / 2.92
CCG	PMA→p-Erk | Fold	0.75	31 / 9	0.080	0.026	2.91 / 1.55
CCG	PMA→p-Erk | Total	0.85	35 / 9	0.001	0.001	4.26 / 2.68
CCG	PMA→p-S6 | Fold	0.72	35 / 9	0.012	0.043	1.87 / 0.96
CCG	PMA→p-S6 | Total	0.72	35 / 9	0.023	0.046	5.15 / 3.87
CCG	SCF→p-Akt | Fold	0.70	58 / 13	0.289	0.029	0.48 / 0.14
CCG	SCF→p-Akt | Total	0.69	47 / 12	0.255	0.046	1.70 / 1.25
CCG	SCF→p-CREB | Total	0.86	7 / 2	0.021	0.222	2.51 / 3.14
CCG	SCF→p-S6 | Fold	0.70	58 / 13	0.035	0.025	1.11 / 0.50
Phosphatase & ROS	H_20_2→p-Akt | Fold	0.79	52 / 12	0.000	0.002	0.83 / 0.37
Phosphatase & ROS	H_20_2→p-Akt | Total	0.70	48 / 12	0.003	0.031	1.19 / 0.83
Phosphatase & ROS	H_20_2→p-Plcγ2 | Total	0.77	48 / 12	0.003	0.005	1.90 / 1.24
Phosphatase & ROS	H_20_2→p-SLP-76 | Fold	0.60	51 / 12	0.036	0.266	0.52 / 0.13
Phosphatase & ROS	H_20_2→p-SLP-76 | Total	0.77	46 / 12	0.001	0.004	2.21 / 1.40

Node/Metrics with a t-test *P* value or Wilcoxon *P* value of ≤ 0.05 and an AUC_{ROC} of ≥ 0.6 are shown

Metrics are defined in the Materials & Methods and shown in Figure 2
