# Supplementary material for: Functional Characterization of FLT3 Receptor Signaling Deregulation in Acute Myeloid Leukemia by Single Cell Network Profiling (SCNP)
Source: PLoS One. 2010 Oct 27;5(10):e13543. doi: 10.1371/journal.pone.0013543 (PMC2965086; doi:10.1371/journal.pone.0013543)
Supplement: Table S5 — Pearson correlations between stratifying nodes for FLT3-ITD vs. FLT3-WT in Study 1. (0.21 MB PDF) [file pone.0013543.s012.pdf]

Table S5. Pearson correlations between stratifying nodes for FLT3-ITD vs FLT3-WT in Study 1.

	Staurosporine & ZVAD→c-PARP | Fold	H$_2$0$_2$ & SCF→p-Erk | Fold	Staurosporine & ZVAD→c-Caspase-8 | Fold	Staurosporine→c-PARP | Fold	Staurosporine & ZVAD→c-Caspase-3 | Fold	Staurosporine→c-Caspase-8 | Fold	Staurosporine→c-Caspase-3 | Fold	H$_2$0$_2$→p-Stat5 | Fold	Etoposide→c-PARP | Fold	Etoposide & ZVAD→c-PARP | Fold	Etoposide→c-Caspase-3 | Fold	M-CSF→p-S6 | Fold	Etoposide→BCL2 | Fold	Thapsigargin→p-CREB | Fold	PMA→p-CREB | Fold	IFNγ→p-Stat5 | Fold	TNFα→p-NFkB-p65 | Fold	H$_2$0$_2$→p-Erk | Fold	IL-6→p-S6 | Fold	IL-6→p-CREB | Fold	G-CSF→p-Erk | Fold	FLT3L→p-S6 | Fold	Etoposide→p-Chk2 | Fold	IL-27→p-Stat5 | Fold	IL-27→p-Stat3 | Fold
Staurosporine & ZVAD→c-PARP | Fold	1.00																								
H$_2$0$_2$ & SCF→p-Erk | Fold	-0.31	1.00																							
Staurosporine & ZVAD→c-Caspase-8 | Fold	0.83	-0.10	1.00																						
Staurosporine→c-PARP | Fold	0.77	-0.14	0.68	1.00																					
Staurosporine & ZVAD→c-Caspase-3 | Fold	0.81	-0.25	0.89	0.61	1.00																				
Staurosporine→c-Caspase-8 | Fold	0.59	0.01	0.72	0.84	0.59	1.00																			
Staurosporine→c-Caspase-3 | Fold	0.73	-0.15	0.69	0.89	0.69	0.84	1.00																		
H$_2$0$_2$→p-Stat5 | Fold	-0.25	0.85	-0.09	-0.25	-0.21	-0.09	-0.26	1.00																	
Etoposide→c-PARP | Fold	0.60	-0.25	0.57	0.60	0.45	0.43	0.41	-0.35	1.00																
Etoposide & ZVAD→c-PARP | Fold	0.56	-0.28	0.53	0.60	0.45	0.44	0.50	-0.29	0.68	1.00															
Etoposide→c-Caspase-3 | Fold	0.45	-0.21	0.51	0.55	0.37	0.45	0.40	-0.40	0.89	0.62	1.00														
M-CSF→p-S6 | Fold	0.26	-0.17	0.30	0.22	0.24	0.30	0.16	-0.02	0.32	0.30	0.20	1.00													
Etoposide→BCL2 | Fold	0.40	-0.06	0.31	0.42	0.15	0.40	0.32	-0.06	0.48	0.32	0.30	0.19	1.00												
Thapsigargin→p-CREB | Fold	0.39	-0.41	0.35	0.30	0.41	0.14	0.19	-0.37	0.54	0.59	0.39	0.51	0.25	1.00											
PMA→p-CREB | Fold	0.37	-0.27	0.34	0.38	0.38	0.16	0.21	-0.38	0.61	0.56	0.49	0.41	0.11	0.87	1.00										
IFNγ→p-Stat5 | Fold	-0.31	0.17	-0.09	-0.35	-0.09	-0.07	-0.09	0.22	-0.51	-0.36	-0.46	-0.17	0.00	-0.44	-0.53	1.00									
TNFα→p-NFkB-p65 | Fold	0.29	-0.44	0.38	0.27	0.39	0.24	0.20	-0.53	0.58	0.60	0.49	0.42	0.23	0.76	0.77	-0.30	1.00								
H$_2$0$_2$→p-Erk | Fold	-0.27	0.94	-0.10	-0.10	-0.27	0.04	-0.09	0.78	-0.28	-0.28	-0.23	-0.36	0.06	-0.48	-0.39	0.32	-0.53	1.00							
IL-6→p-S6 | Fold	-0.07	-0.07	-0.08	-0.19	0.00	-0.33	-0.27	-0.11	0.06	0.18	0.02	0.17	0.06	0.48	0.38	0.00	0.40	-0.09	1.00						
IL-6→p-CREB | Fold	0.41	-0.14	0.57	0.40	0.57	0.26	0.33	-0.27	0.53	0.39	0.51	0.25	-0.10	0.53	0.68	-0.29	0.61	-0.26	0.27	1.00					
G-CSF→p-Erk | Fold	0.13	-0.13	0.27	0.16	0.00	0.09	0.03	-0.39	0.51	0.31	0.64	0.21	0.12	0.29	0.45	-0.32	0.48	-0.19	0.14	0.51	1.00				
FLT3L→p-S6 | Fold	-0.41	0.20	-0.28	-0.57	-0.05	-0.51	-0.39	0.16	-0.33	-0.26	-0.26	-0.12	-0.52	-0.12	-0.12	0.30	-0.07	0.12	0.37	0.11	-0.19	1.00			
Etoposide→p-Chk2 | Fold	-0.45	-0.05	-0.40	-0.40	-0.30	-0.29	-0.24	0.10	-0.75	-0.31	-0.73	-0.30	-0.47	-0.35	-0.44	0.47	-0.36	0.02	-0.08	-0.34	-0.48	0.19	1.00		
IL-27→p-Stat5 | Fold	-0.24	0.39	-0.13	-0.45	-0.20	-0.44	-0.47	0.40	-0.09	-0.11	-0.27	0.14	0.03	0.04	0.11	0.30	0.00	0.33	0.41	-0.06	0.06	0.34	0.00	1.00	
IL-27→p-Stat3 | Fold	-0.42	0.26	-0.25	-0.56	-0.22	-0.57	-0.55	0.27	-0.10	-0.16	-0.18	0.20	-0.32	0.13	0.22	0.23	0.11	0.10	0.41	0.18	0.11	0.58	0.10	0.81	1.00

Blue = negative correlation, yellow = no relationship, green = positive correlation

Correlation (R) Values between FLT3-WT vs FLT3-ITD stratifying nodes demonstrate correlated nodes/pathways (IL-27 → p-Stat3 | Fold, IL-27 → p-Stat5, | Fold: R=.81) and non-correlated nodes/pathways (IL-27 → p-Stat5, | Fold , FLT3L → p-S6 | Total: R=.19) suggesting that distinct pathways may provide independent information in characterizing the biology of FLT3-ITD and FLT3-WT AML samples. An R value of 0 indicates no correlation, positive R values (0-1) indicate positive correlation and negative R values (-1 to 0) indicate negative correlation. Correlations based on 25 donors with data for all nodes.
