# Supplementary material for: Functional Characterization of FLT3 Receptor Signaling Deregulation in Acute Myeloid Leukemia by Single Cell Network Profiling (SCNP)
Source: PLoS One. 2010 Oct 27;5(10):e13543. doi: 10.1371/journal.pone.0013543 (PMC2965086; doi:10.1371/journal.pone.0013543)
Supplement: Table S6 — Combinations/pairs of nodes from linear regression analysis improve stratification of FLT3-ITD and WT samples in Study 1. (0.08 MB PDF) [file pone.0013543.s013.pdf]

Table S6. Combinations / pairs of nodes from linear regression analysis improve stratification of FLT3-ITD and WT samples in Study 1.

Node 1	Node 2	Num WTs/ITDs	AUC Model	AUC Node1 Alone	AUC Node2 Alone	Node1 p-Value	Node2 p-Value
Staurosporine & ZVAD→c-Caspase-8 | Fold	SCF→p-Stat5 | Total	15 / 14	0.99	0.90	0.85	0.048	0.044
IL-6→p-Stat5 | Total	FLT3L→p-S6 | Total	17 / 15	0.98	0.84	0.80	0.028	0.024
Staurosporine & ZVAD→c-Caspase-8 | Fold	M-CSF→p-Stat5 | Total	15 / 14	0.98	0.90	0.84	0.032	0.040
Staurosporine & ZVAD→c-Caspase-8 | Fold	IGF-1→p-Stat5 | Total	15 / 14	0.98	0.90	0.85	0.047	0.047
Staurosporine & ZVAD→c-Caspase-8 | Fold	H_2O_2→p-Stat5 | Fold	14 / 14	0.97	0.89	0.84	0.032	0.046
p-Stat5 | Basal	FLT3L→p-S6 | Total	17 / 15	0.97	0.85	0.80	0.029	0.030
Staurosporine→c-Caspase-8 | Total	IL-27→p-Erk | Total	15 / 14	0.97	0.87	0.71	0.014	0.021
Staurosporine & ZVAD→c-Caspase-3 | Total	FLT3L→p-CREB | Total	15 / 14	0.97	0.89	0.81	0.039	0.021
Staurosporine & ZVAD→c-Caspase-8 | Fold	p-Stat5 | Basal	15 / 14	0.97	0.90	0.83	0.040	0.044
IL-27→p-Stat5 | Total	Staurosporine→c-Caspase-8 | Fold	15 / 14	0.97	0.83	0.85	0.036	0.049
H_2O_2 & SCF→p-Erk | Fold	Etoposide & ZVAD→p-Chk2+, c-PARP + | Quad	14 / 14	0.97	0.91	0.77	0.035	0.044
Staurosporine→c-Caspase-8 | Total	TNFα→p-NFkB-p65 | Fold	15 / 14	0.97	0.87	0.69	0.008	0.022
SCF→p-Stat5 | Total	Staurosporine→c-Caspase-8 | Fold	15 / 14	0.97	0.85	0.85	0.030	0.028
M-CSF→p-Stat5 | Total	Staurosporine→c-Caspase-8 | Fold	15 / 14	0.97	0.84	0.85	0.033	0.030
Staurosporine→c-Caspase-8 | Total	FLT3L→p-S6 | Total	15 / 14	0.97	0.87	0.80	0.015	0.034
FLT3L→p-S6 | Total	IFNγ→p-Stat5 | Total	17 / 15	0.96	0.80	0.77	0.014	0.017
IL-27→p-Stat5 | Total	FLT3L→p-S6 | Total	17 / 15	0.96	0.85	0.80	0.014	0.024
IL-6→p-Stat5 | Total	Etoposide & ZVAD→p-Chk2+, c-PARP - | Quad	14 / 14	0.96	0.79	0.78	0.027	0.018
Staurosporine & ZVAD→c-Caspase-3 | Fold	H_2O_2→p-Stat5 | Fold	14 / 14	0.96	0.85	0.84	0.028	0.022
IL-6→p-Stat5 | Total	Etoposide & ZVAD→c-PARP | Total	14 / 14	0.96	0.79	0.80	0.028	0.013
H_2O_2 & SCF→p-Erk | Fold	Etoposide & ZVAD→p-Chk2+, c-PARP - | Quad	14 / 14	0.96	0.91	0.78	0.027	0.037
Staurosporine & ZVAD→c-Caspase-8 | Fold	p-Stat5 | Total	15 / 14	0.96	0.90	0.88	0.037	0.037
Staurosporine & ZVAD→c-Caspase-3 | Total	TNFα→p-NFkB-p65 | Fold	15 / 14	0.96	0.89	0.69	0.012	0.043
SCF→p-Stat5 | Total	p-SLP-76 | Total	13 / 14	0.96	0.84	0.76	0.013	0.018
Staurosporine→c-Caspase-8 | Fold	ABCG2 | PercentPos	14 / 13	0.96	0.85	0.71	0.043	0.032
M-CSF→p-Stat5 | Total	FLT3L→p-S6 | Total	17 / 15	0.96	0.85	0.80	0.014	0.026
SCF→p-Stat5 | Total	IL-27→p-Erk | Total	17 / 15	0.96	0.87	0.71	0.006	0.028
IGF-1→p-Stat5 | Total	FLT3L→p-S6 | Total	17 / 15	0.96	0.85	0.80	0.017	0.034
IL-10→p-Stat5 | Total	FLT3L→p-S6 | Fold	17 / 15	0.96	0.87	0.72	0.042	0.045
Staurosporine→c-Caspase-8 | Total	Etoposide & ZVAD→c-PARP | Total	14 / 14	0.96	0.86	0.80	0.024	0.031
Staurosporine→c-Caspase-8 | Total	Etoposide→c-PARP | Total	14 / 14	0.96	0.86	0.82	0.025	0.037
H_2O_2 & SCF→p-Erk | Fold	G-CSF→p-S6 | Total	14 / 14	0.96	0.91	0.65	0.026	0.039
EPO→p-Stat5 | Total	H_2O_2 & SCF→p-Erk | Total	14 / 14	0.96	0.85	0.72	0.040	0.035
H_2O_2 & SCF→p-Erk | Fold	SCF→p-CREB | Total	14 / 14	0.96	0.91	0.77	0.032	0.042
Staurosporine→c-Caspase-8 | Total	Etoposide→c-Caspase-3 | Total	12 / 14	0.96	0.86	0.82	0.036	0.049
Staurosporine→c-Caspase-8 | Total	CD40L→p-p38 | Total	15 / 14	0.96	0.87	0.67	0.016	0.032
Staurosporine→c-Caspase-8 | Total	IL-6→p-CREB | Fold	15 / 14	0.96	0.87	0.73	0.010	0.035
p-Stat5 | Basal	Staurosporine→c-Caspase-8 | Fold	15 / 14	0.96	0.83	0.85	0.037	0.042
Staurosporine & ZVAD→c-Caspase-8 | Fold	EPO→p-Stat5 | Total	15 / 14	0.96	0.90	0.85	0.031	0.045
Staurosporine & ZVAD→c-Caspase-8 | Fold	IL-10→p-Stat5 | Total	15 / 14	0.96	0.90	0.86	0.022	0.047
Staurosporine→c-Caspase-8 | Fold	IFNα→p-Stat5 | Total	15 / 14	0.96	0.85	0.78	0.032	0.050
p-Stat5 | Basal	M-CSF→p-S6 | Fold	17 / 15	0.96	0.89	0.77	0.013	0.032
M-CSF→p-Stat5 | Total	p-SLP-76 | Basal	13 / 14	0.96	0.82	0.76	0.013	0.017
IGF-1→p-Stat5 | Total	p-SLP-76 | Basal	13 / 14	0.96	0.85	0.76	0.020	0.025
IL-10→p-Stat5 | Total	Etoposide→c-PARP | Fold	14 / 14	0.95	0.86	0.83	0.022	0.019
Staurosporine & ZVAD→c-Caspase-3 | Total	H_2O_2→p-Stat5 | Fold	14 / 14	0.95	0.88	0.84	0.014	0.023
IL-27→p-Stat5 | Total	H_2O_2 & SCF→p-Erk | Total	14 / 14	0.95	0.82	0.72	0.009	0.023
IL-10→p-Stat5 | Total	H_2O_2 & SCF→p-Erk | Total	14 / 14	0.95	0.86	0.72	0.007	0.029
IL-10→p-Stat5 | Total	Etoposide & ZVAD→c-PARP | Total	14 / 14	0.95	0.86	0.80	0.029	0.034
Staurosporine→c-Caspase-8 | Total	Etoposide→c-PARP | Fold	14 / 14	0.95	0.86	0.83	0.019	0.040
H_2O_2 & SCF→p-Erk | Fold	Etoposide & ZVAD→p-Chk2-, c-PARP + | Quad	14 / 14	0.95	0.91	0.74	0.016	0.042
SCF→p-Stat5 | Total	FLT3L→p-S6 | Total	17 / 15	0.95	0.87	0.80	0.011	0.033
EPO→p-Stat5 | Total	M-CSF→p-S6 | Fold	17 / 15	0.95	0.87	0.77	0.012	0.036
SCF→p-Stat5 | Total	Etoposide→c-Caspase-3 | Total	12 / 14	0.95	0.85	0.82	0.021	0.039

Filtered on AUC Model > 0.95 , p<.05.

Summary of results from logistic regression models which include 2 nodes at a time from the list of top ranking nodes to model FLT3 mutation status. The columns show the number of donors from each group, the AUC for the model, and the AUCs for each of the nodes by themselves and the p-values (coeff = 0.0) for coefficient for each of the nodes.
