# Supplementary material for: Functional Characterization of FLT3 Receptor Signaling Deregulation in Acute Myeloid Leukemia by Single Cell Network Profiling (SCNP)
Source: PLoS One. 2010 Oct 27;5(10):e13543. doi: 10.1371/journal.pone.0013543 (PMC2965086; doi:10.1371/journal.pone.0013543)
Supplement: Table S7 — Clinical characteristics of cytogenetically normal (CN) AML patient samples. (0.07 MB PDF) [file pone.0013543.s014.pdf]

Table S7. Clinical characteristics of cytogenetically normal (CN) AML patient samples.

Study 1							
**Characteristic**		**FLT3-ITD [count =10]**		**FLT3-WT [count =7]**		**ALL [count=17]**	
**Age**		**Median** 48.0	**Range** 24-71	**Median** 50.0	**Range** 39-75	**Median** 48.0	**Range** 25-75
**Age Category**	<60	80.0 (%)	8	71.4 (%)	5	76.5 (%)	13
	>60	20.0 (%)	2	28.6 (%)	2	23.5 (%)	4
**Gender**	M	40.0 (%)	4	14.3 (%)	1	29.4 (%)	5
	F	60.0 (%)	6	85.7 (%)	6	70.6 (%)	12
**Race**	black	0.0 (%)	0	14.3 (%)	1	5.9 (%)	1
	white	70.0 (%)	7	28.6 (%)	2	52.9 (%)	9
	hispanic	0.0 (%)	0	0.0 (%)	0	0.0 (%)	0
	asian	30.0 (%)	3	42.9 (%)	3	35.3 (%)	6
	unknown	0.0 (%)	0	14.3 (%)	1	5.9 (%)	1
**FAB**	m0	0.0 (%)	0	0.0 (%)	0	0.0 (%)	0
	m1	10.0 (%)	1	28.6 (%)	2	17.6 (%)	3
	m2	20.0 (%)	2	0.0 (%)	0	11.8 (%)	2
	m4	30.0 (%)	3	28.6 (%)	2	29.4 (%)	5
	m5	10.0 (%)	1	14.3 (%)	1	11.8 (%)	2
	m6	0.0 (%)	0	0.0 (%)	0	0.0 (%)	0
	m7	0.0 (%)	0	0.0 (%)	0	0.0 (%)	0
	unknown	30.0 (%)	3	28.6 (%)	2	29.4 (%)	5
**FLT3-ITD**	Pos	100.0 (%)	10	0.0 (%)	0	58.8 (%)	10
	Neg	0.0 (%)	0	100.0 (%)	7	41.2 (%)	7
	NA	0.0 (%)	0	0.0 (%)	0	0.0 (%)	0
**FLT3 TKD**	Neg	100.0 (%)	10	100.0 (%)	7	100.0 (%)	17
	Pos	0.0 (%)	0	0.0 (%)	0	0.0 (%)	0
**Secondary AML**	Yes	0.0 (%)	0	0.0 (%)	0	0.0 (%)	0
	No	100.0 (%)	10	100.0 (%)	7	100.0 (%)	17
**Therapy**	IA+Zarnestra	0.0 (%)	0	0.0 (%)	0	0.0 (%)	0
	IDA+HDAC	0.0 (%)	0	0.0 (%)	0	0.0 (%)	0
	Other	0.0 (%)	0	0.0 (%)	0	0.0 (%)	0
	Standard 3+7	100.0 (%)	10	100.0 (%)	7	100.0 (%)	17
**Induction Response**	CR	40.0 (%)	4	28.6 (%)	2	35.3 (%)	6
	NR	60.0 (%)	6	71.4 (%)	5	64.7 (%)	11
**CR Duration (Weeks)**		**Median** 39.0	**Range** 15-66	**Median** 96.0	**Range** 96	**Median** 47.0	**Range** 15-96

Study 2							
**Characteristic**		**FLT3-ITD [count =10]**		**FLT3-WT [count = 24]**		**ALL [count=34]**	
**Age**		**Median** 52.0	**Range** 37-69	**Median** 55.7	**Range** 27-79	**Median** 55.7	**Range** 27-79
**Age Category**	<60	90.0 (%)	9	83.3 (%)	20	85.3 (%)	29
	>60	10.0 (%)	1	16.7 (%)	4	14.7 (%)	5
**Gender**	M	30.0 (%)	3	45.8 (%)	11	41.2 (%)	14
	F	70.0 (%)	7	54.2 (%)	13	58.8 (%)	20
**Race**	black	20.0 (%)	2	8.3 (%)	2	11.8 (%)	4
	white	30.0 (%)	3	33.3 (%)	8	32.4 (%)	11
	hispanic	10.0 (%)	1	4.2 (%)	1	5.9 (%)	2
	asian	0.0 (%)	0	4.2 (%)	1	2.9 (%)	1
	unknown	40.0 (%)	4	50.0 (%)	12	47.1 (%)	16
**FAB**	m0	0.0 (%)	0	0.0 (%)	0	0.0 (%)	0
	m1	50.0 (%)	5	8.3 (%)	2	20.6 (%)	7
	m2	30.0 (%)	3	50.0 (%)	12	44.1 (%)	15
	m4	10.0 (%)	1	16.7 (%)	4	14.7 (%)	5
	m5	10.0 (%)	1	16.7 (%)	4	14.7 (%)	5
	m6	0.0 (%)	0	4.2 (%)	1	2.9 (%)	1
	m7	0.0 (%)	0	0.0 (%)	0	0.0 (%)	0
	unknown	0.0 (%)	0	4.2 (%)	1	2.9 (%)	1
**FLT3-ITD**	Pos	100.0 (%)	10	0.0 (%)	0	38.2 (%)	13
	Neg	0.0 (%)	0	100.0 (%)	23	29.4 (%)	10
	NA	0.0 (%)	0	0.0 (%)	0	0.0 (%)	0
**FLT3 TKD**	Neg	80.0 (%)	8	87.5 (%)	21	85.3 (%)	29
	Pos	20.0 (%)	2	12.5 (%)	3	14.7 (%)	5
**Secondary AML**	Yes	20.0 (%)	2	20.8 (%)	5	20.6 (%)	7
	No	80.0 (%)	8	79.2 (%)	19	79.4 (%)	27
**Therapy**	IA+Zarnestra	50.0 (%)	5	50.0 (%)	12	50.0 (%)	17
	IDA+HDAC	10.0 (%)	1	29.2 (%)	7	23.5 (%)	8
	Other	40.0 (%)	4	20.8 (%)	5	26.5 (%)	9
	Standard 3+7	0.0 (%)	0	0.0 (%)	0	0.0 (%)	0
**Induction Response**	CR	70.0 (%)	7	83.3 (%)	20	79.4 (%)	27
	NR	30.0 (%)	3	16.7 (%)	4	20.6 (%)	7
**CR Duration (Weeks)**		**Median** 27.0	**Range** 4-142	**Median** 55.0	**Range** 6-169	**Median** 51.0	**Range** 4-169
