# Supplementary material for: Functional Characterization of FLT3 Receptor Signaling Deregulation in Acute Myeloid Leukemia by Single Cell Network Profiling (SCNP)
Source: PLoS One. 2010 Oct 27;5(10):e13543. doi: 10.1371/journal.pone.0013543 (PMC2965086; doi:10.1371/journal.pone.0013543)
Supplement: Table S8 — Clinical characteristics of FLT3-WT vs. FLT3-ITD signaling outliers (Study 2). (0.06 MB PDF) [file pone.0013543.s015.pdf]

Table S8. Clinical characteristics of FLT3-WT vs FLT3-ITD signaling outliers (Study 2).

Donor ID	FAB	Sex	Age at Dx	WBC cells/mm^3), PB	BM % Blasts	Secondary AML	Cytogenetic Group	FLT3-ITD	FLT-TKD (D835)	Response	Response Category	CR Duration (weeks)	# Node/metrics with Donor as outlier
MD1-48	M1	F	36.7	5	78	No	INTERMEDIATE	NEG	NEG	CR	CR Relpase	53	1
MD2-07	M5	M	46.0	53	78	No	INTERMEDIATE	POS	NEG	CR	CR Relpase	27	3
MD2-13	M4	M	49.4	12	82	No	UNFAVORABLE	NEG	NEG	CR	CR Relpase	13	1
MD2-14	M1	F	48.3	82	92	No	INTERMEDIATE	POS	NEG	CR	CR Relpase	26	5
**MD2-22**	**M1**	**F**	**36.8**	**46**	**84**	**No**	**INTERMEDIATE**	**POS**	**NEG**	**CR**	**Continuing CR**	**142**	**16**
MD2-26	M4	F	58.4	29	20	No	UNFAVORABLE	NEG	NEG	CR	CR Relpase	20	1
MD2-43	M2	F	68.6	12	64	Yes	INTERMEDIATE	POS	NEG	NR	Refractory	NA	2
MD3-18	M2	F	43.7	18	60	No	FAVORABLE	NEG	NEG	CR	CR Relpase	68	1
MD3-19	M2	F	56.5	2	33	Yes	INTERMEDIATE	NEG	NEG	CR	CR Relpase	10	1
MD3-22	M2	M	58.4	9	69	No	INTERMEDIATE	POS	POS	CR	Continuing CR	106	1
MD3-29	M1	M	27.0	16	84	No	INTERMEDIATE	NEG	NEG	CR	Ongoing CR	44	2
MD3-32	M2	F	34.9	27	65	No	UNFAVORABLE	POS	NEG	CR	CR Relpase	11	1
MD3-34	M4	F	55.7	199	79	No	INTERMEDIATE	POS	POS	CR	CR Relpase	4	1
MD3-49	M2	M	72.3	19	20	No	INTERMEDIATE	NEG	NEG	CR	Continuing CR	211	2
MD3-65	M2	M	72.6	36	85	No	INTERMEDIATE	NEG	POS	CR	Continuing CR	102	1
MD3-66	M1	F	43.3	124	91	No	INTERMEDIATE	POS	NEG	CR	CR Relpase	48	1

Clinical outlier MD2-22, a FLT3-ITD sample with remission >2 years, signals in many nodes like a FLT3-WT sample.
Clinical Characteristics of MD2-22 are shown above
MD2-22 is an outlier relative to other FLT3-ITD samples for 16 signaling node/metrics

CR: Complete response; NR: Non-complete response;
Continuing CR: CR duration > 2 years; Ongoing CR: CR duration < 2 years; CR Relapse-relapse following CR
