# Supplementary material for: Functional Characterization of FLT3 Receptor Signaling Deregulation in Acute Myeloid Leukemia by Single Cell Network Profiling (SCNP)
Source: PLoS One. 2010 Oct 27;5(10):e13543. doi: 10.1371/journal.pone.0013543 (PMC2965086; doi:10.1371/journal.pone.0013543)
Supplement: Table S9 — List of modulators, reagents and technical conditions. (0.08 MB PDF) [file pone.0013543.s016.pdf]

Table S9A. List of modulators and technical conditions.

Modulator	Final Concentration	Modulator Treatment Duration	Manufacturer (Location)
Ara-C	0.5 µg/mL	24 hours	Sigma Aldrich (St Louis, MO)
CD40L	0.5 µg/mL	7.5 mins and 15 mins	R&D (Minneapolis, MN)
Daunorubicin	100 ng/mL	24 hours	Sigma Aldrich (St Louis, MO)
EPO	1 IU/mL	15 mins	R&D (Minneapolis, MN)
Etoposide	30 µg/mL	24 hours	Sigma Aldrich (St Louis, MO)
FCS	1.0%	various	HyClone (Waltham, MA)
FLT3L	50 ng/mL	15 mins	eBiosciences (San Diego, CA)
G-CSF*	50 ng/mL	15 mins	R&D (Minneapolis, MN)
G-CSF*	50 ng/mL	15 mins	Pepro (Rocky Hill, NJ)
GM-CSF	2 ng/mL	15 mins	BD (San Jose, CA)
H_2O_2	2.33 mM	15 mins	JT Baker (Phillipsburg, NJ)
IFNα	10000 IU/mL	15 mins	Schering (Kenilworth, NJ)
IFNγ	5 ng/mL	15 mins	BD (San Jose, CA)
IGF-1	6.66 ng/mL	15 mins	R&D (Minneapolis, MN)
IL-10	25 ng/mL	15 mins	BD (San Jose, CA)
IL-27	50 ng/mL	15 mins	R&D (Minneapolis, MN)
IL-3	50 ng/mL	15 mins	BD (San Jose, CA)
IL-4	5 ng/mL	15 mins	BD (San Jose, CA)
IL-6	25 ng/mL	15 mins	R&D (Minneapolis, MN)
LPS	1 µg/mL	7.5 mins	Sigma Aldrich (St Louis, MO)
M-CSF	2 ng/mL	15 mins	R&D (Minneapolis, MN)
PMA	400 nM	15 mins	Sigma Aldrich (St Louis, MO)
SCF	20 ng/mL	15 mins	R&D (Minneapolis, MN)
SDF-1α	2 ng/mL	3 mins	R&D (Minneapolis, MN)
Staurosporine	2.33 µg/mL	6 hours	Sigma Aldrich (St Louis, MO)
Thapsigargin	1 µM	15 mins	EMD Biosciences (Darmstadt, Germany)
TNFα	20 ng/mL	7.5 mins	BD (San Jose, CA)
ZVAD	100µM	24 hours	R&D (Minneapolis, MN)

* Products displayed similar bioactivity

Table S9B. Antibodies and reagents used.

Antibody	Species & Isotype	Manufacturer (Location)	Clone
ABCG2	Mouse IgG_{2b}	R&D (Minneapolis, MN)	5D3
BCL-2	Mouse $IgG_{1,\,k}$	BD (San Jose, CA)	100
CD11b	Mouse IgG_1	Beckman (Miami, FL)	Bear1
CD33†	Mouse IgG_1	Beckman (Miami, FL)	D3HL60.251
CD33†	Mouse IgG_1	BD (San Jose, CA)	P67.6
CD34	Mouse IgG_1	BD (San Jose, CA)	8G12
CD40	Mouse IgG1, k	BD (San Jose, CA)	5C3
CD45	Mouse IgG_1	Invitrogen (Carlsbad, CA)	HI30
cKit	Mouse IgG_1	R&D (Minneapolis, MN)	47233
c-Caspase 3	Rabbit IgG	BD (San Jose, CA)	C92-605
c-Caspase 8 (Asp391)	Rabbit IgG	CST (Danvers, MA)	18C8
c-PARP(Asp214)	Mouse IgG_1, k	BD (San Jose, CA)	F21-852
Control Ig	Mouse IgG1	eBiosciences (San Diego, CA)	unknown
Control Ig	Mouse IgG_{2a}, k	BD (San Jose, CA)	X39
Control Ig	Rat IgG1	MBL (Woburn, MA)	1H5
Control Ig	Mouse IgG_{2b}	R&D (Minneapolis, MN)	13303
Control Ig	Mouse IgG_1, k	BD (San Jose, CA)	MOPC-21
CXCR4†	Mouse IgG2a, k	BD (San Jose, CA)	12G5
CXCR4†	Rat IgG1	MBL (Woburn, MA)	A145
Cytochrome C	Mouse IgG_{2b}, k	BD (San Jose, CA)	7H8.2C12
EPO-R	Mouse IgG2b	R&D (Minneapolis, MN)	38409
FLT3 Receptor†	Mouse IgG1	R&D (Minneapolis, MN)	66903
FLT3 Receptor†	Mouse IgG_1	eBiosciences (San Diego, CA)	BV10A4H2
Goat anti-rabbit secondary	Goat IgG	Invitrogen (Carlsbad, CA)	Polyclonal
M-CSFR	Mouse IgG1	R&D (Minneapolis, MN)	61708
MRP-1	Mouse IgG_1	R&D (Minneapolis, MN)	QCRL
p-Akt (S473)	Rabbit IgG	CST (Danvers, MA)	193H12
p-Chk2 (T68)	Rabbit IgG	CST (Danvers, MA)	Polyclonal
p-CREB (pS133)†	Rabbit IgG	CST (Danvers, MA)	87G3
p-CREB (pS133)†	Mouse IgG_1, k	BD (San Jose, CA)	J151-21
p-Erk 1/2 (T202/204)	Mouse IgG_1	BD (San Jose, CA)	20A
p-Lck (Y505)	Mouse IgG_1	BD (San Jose, CA)	4/Lck-Y505
p-NFkB p65 (pS529)	Mouse IgG_{2b}, k	BD (San Jose, CA)	K10-895.12.50
p-p38 MAPK (pT180/pY182)	Mouse IgG_1	BD (San Jose, CA)	36/p38 (pT180/pY182)
p-PLCγ2 (Y759)	Mouse IgG_1, k	BD (San Jose, CA)	K86-689.37
p-S6 (S235/236)	Rabbit IgG	CST (Danvers, MA)	2F9
p-SLP-76 (pY128)	Mouse IgG_1, k	BD (San Jose, CA)	J141-668.36.58
p-Stat1 (pY701)	Mouse IgG_{2a}	BD (San Jose, CA)	4a
p-Stat3 (pY705)	Mouse IgG_{2a}, k	BD (San Jose, CA)	4/P-STAT3
p-Stat5 (pY694)	Mouse IgG_1	BD (San Jose, CA)	47
p-Stat6 (pY641)	Mouse IgG_{2a}	BD (San Jose, CA)	18/P-Stat-6
TNF-R1	Mouse IgG_{2a}	Beckman (Miami, FL)	H398
Non-Antibody Stains	n/a	Manufacturer (Location)	n/a
Amine Aqua Viability Dye	n/a	Invitrogen (Carlsbad, CA)	n/a
Streptavidin-Qdot 605	n/a	Invitrogen (Carlsbad, CA)	n/a

† Changes reflect reagents optimization for Training study 2
